# Production of a reference transcriptome and transcriptomic database (PocilloporaBase) for the cauliflower coral, *Pocillopora damicornis*

**DOI:** 10.1186/1471-2164-12-585

**Published:** 2011-11-29

**Authors:** Nikki Traylor-Knowles, Brian R Granger, Tristan J Lubinski, Jignesh R Parikh, Sara Garamszegi, Yu Xia, Jarrod A Marto, Les Kaufman, John R Finnerty

**Affiliations:** 1 Department of Biology, Stanford University, Hopkins Marine Station, Ocean View Blvd., Pacific Grove, CA, 93950, USA; 2 Department of Biology, Boston University, 5 Cummington Street, Boston, MA 02215, USA; 3 Bioinformatics Program, Boston University, 24 Cummington Street, Boston, MA 02215, USA; 4 Department of Chemistry, Boston University, 24 Cummington Street, Boston, MA 02215, USA; 5 Department of Biomedical Engineering, Boston University, 24 Cummington Street, Boston, MA 02215, USA; 6 Department of Cancer Biology and Blais Proteomics Center, Dana Farber Cancer Institute, 44 Binney Street, Boston, MA 02115, USA; 7 Department of Biological Chemistry and Molecular Pharmacology, 44 Binney Street, Harvard Medical School, Boston, MA 02115, USA

## Abstract

**Background:**

Motivated by the precarious state of the world's coral reefs, there is currently a keen interest in coral transcriptomics. By identifying changes in coral gene expression that are triggered by particular environmental stressors, we can begin to characterize coral stress responses at the molecular level, which should lead to the development of more powerful diagnostic tools for evaluating the health of corals in the field. Furthermore, the identification of genetic variants that are more or less resilient in the face of particular stressors will help us to develop more reliable prognoses for particular coral populations. Toward this end, we performed deep mRNA sequencing of the cauliflower coral, *Pocillopora damicornis*, a geographically widespread Indo-Pacific species that exhibits a great diversity of colony forms and is able to thrive in habitats subject to a wide range of human impacts. Importantly, *P. damicornis *is particularly amenable to laboratory culture. We collected specimens from three geographically isolated Hawaiian populations subjected to qualitatively different levels of human impact. We isolated RNA from colony fragments ("nubbins") exposed to four environmental stressors (heat, desiccation, peroxide, and hypo-saline conditions) or control conditions. The RNA was pooled and sequenced using the 454 platform.

**Description:**

Both the raw reads (n = 1, 116, 551) and the assembled contigs (n = 70, 786; mean length = 836 nucleotides) were deposited in a new publicly available relational database called PocilloporaBase http://www.PocilloporaBase.org. Using BLASTX, 47.2% of the contigs were found to match a sequence in the NCBI database at an E-value threshold of ≤.001; 93.6% of those contigs with matches in the NCBI database appear to be of metazoan origin and 2.3% bacterial origin, while most of the remaining 4.1% match to other eukaryotes, including algae and amoebae.

**Conclusions:**

*P. damicornis *now joins the handful of coral species for which extensive transcriptomic data are publicly available. Through PocilloporaBase http://www.PocilloporaBase.org, one can obtain assembled contigs and raw reads and query the data according to a wide assortment of attributes including taxonomic origin, PFAM motif, KEGG pathway, and GO annotation.

## Background

Over the long-term, populations can respond to stressful environmental conditions via adaptive evolution. However, over the short-term, organisms under stress must alter their physiology or behavior, and doing so commonly involves changes in gene expression. Therefore, if we are to understand how organisms respond to stress, we must understand how stress alters gene expression.

In recent years, a number of microarray studies have been undertaken to reconstruct the gene expression profiles of corals under stress [1-6]. However, the chip-based approaches used to date have not been able to evaluate the full scope of the stress response because only a fraction of potential transcripts have been represented on the chips. In the absence of a tiling array, whose production must await the sequencing and complete assembly of a coral genome, next-generation sequencing technologies are the only available approach for characterizing the full scope of the transcriptional response to stress. Currently, there is no complete coral genome available, and the most closely related genome that is publicly available is that of the starlet sea anemone *Nematostella vectensis *[7].

In the environment, corals are exposed to a variety of natural and anthropogenic stressors. Once the stressors reach a tipping point, dramatic physiological changes can occur very abruptly, including bleaching, which involves the expulsion of all symbiotic zooxanthellae [8-15]. While the loss of symbionts may be adaptive in the short-term, once bleaching occurs, if a coral colony continues to experience stressful conditions, it will rapidly succumb--scleractinian corals cannot persist indefinitely as heterotrophs. Therefore, a critical component of coral resilience is their ability to stave off bleaching through molecular stress-response mechanisms.

To date, the identification of "stress response genes" in corals and other cnidarians has been based largely on homology to functionally characterized genes in other animals (*e.g*., [16]). This approach has validity, as there is accumulating evidence that many cnidarian stress responses are likely to be largely homologous to those of triploblastic animals. However, given the long history of the Cnidaria as an independent evolutionary lineage and the many unique aspects of their biology, the cnidarian stress response repertoire is also certain to differ in key respects from that of other animals.

Fortunately, the development of genomic, transcriptomic, and proteomic approaches has facilitated the *de novo *identification of cnidarian stress response genes. Over the last few years, a number of scleractinian transcriptomic datasets have been made available (Table 1), including EST collections produced by Sanger sequencing (e.g., *Montastrea faveolata, Acropora palmata, Acropora millepora; *[4,17-19]) or "next generation" pyrosequencing (e.g., *Acropora millepora, Acropora hyacinthus, Porites compressa*, and *Porites asteroides; *[20]). Many of these projects have focused on the effects of heat stress on the physiology of the corals and their symbiotic algae, *Symbiodinium *[2,17,19-21].

**Table 1:** Published transcriptomic datasets for scleractinians

Species	Major lineage	Sequencing platform	Source of RNA	Reads	Avg. Length (nt)	Yield (Mb)	**Ref**.
*Acropora millepora*	Complexa	454 GS-Flx	larvae; heat-stressed larvae; larvae treated with settlement inducer	628, 649	232	145.8	[20,37]
*Acropora palmata*	Complexa	Sanger	developmental stages, incl. adults; with and without symbionts	14, 588	500 estimated	7.29 estimated	[17]
*Montastrea faveolata*	Robusta	Sanger	developmental stages, incl. adults; with and without symbionts	3, 854	500 estimated	1.93 estimated	[17]
*Pocillopora damicornis*	Robusta	454	adult colonies subject to a battery of stressors	955, 105	379	362.0	This study.

**Figure 1:**
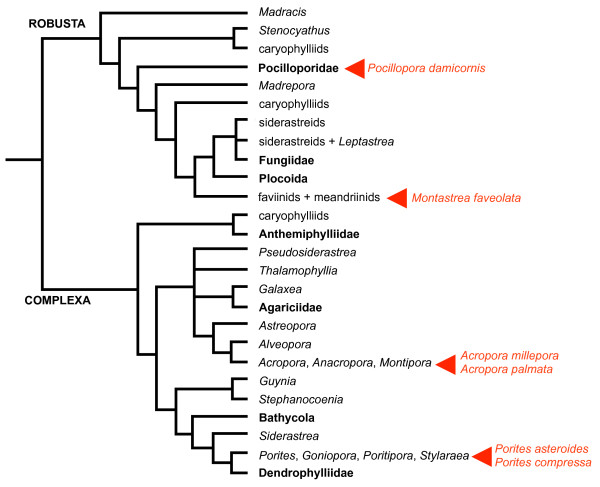
**Coral Phylogeny**. A phylogenetic supertree of stony corals (modified from [22]). *P. damicornis *is nested within the Robusta clade. Red arrowheads indicate taxa for which extensive transcriptomic data are publicly available.

As part of this global effort to characterize the coral stress response at the level of gene expression, we have produced a reference transcriptome for the cauliflower coral, *Pocillopora damicornis *(Linnaeus, 1758) using adult colonies collected in Hawaii. We chose *P. damicornis *because of its wide distribution across the Indo-Pacific, its recognized ability to tolerate environmental stressors that prove insurmountable to many other species, and the relative ease of maintaining it in the laboratory setting. In addition, with respect to transcriptomic data, *P. damicornis *represents a relatively under-sampled branch of the "robust corals" clade (Figure 1) [22], so data from this species is phylogenetically complementary to existing data from *Acropora *and *Porites *(two genera of complex corals) and *Montastrea *(a distantly related robust coral). RNA was isolated from colony nubbins that had been exposed to one of four different biologically relevant stressors (heat, desiccation, peroxide, and hypo-saline conditions) in addition to unstressed, control nubbins. We sampled colonies from three geographically isolated sites that are subjected to qualitatively different degrees of anthropogenic disturbance. The raw data, as well as assembled contigs, have been placed in a publicly accessible, BLAST-searchable relational database called PocilloporaBase.

## Construction and content

### PocilloporaBase Database Construction

PocilloporaBase is a relational database constructed in PostgreSQL (version 8.4.4). It houses the *P. damicornis *contigs generated in this study in addition to the results from a number of bioinformatics analyses performed on these contigs. The database structure and entity relationships are depicted in Additional File 1.

The database comprises thirteen tables, eight entity tables and five relations tables. The "Contigs" table houses output from the assembly including the nucleotide sequence of each contig and other key features of the contig, including its length in nucleotides, possible start sites, the total number of reads used to build the contig, and the average sequencing coverage of each nucleotide position within the contig. Through the "Hmm" table, each contig ID (CID) is linked to 0, 1 or more protein family IDs (PFID) based on a search of Pfam (an HMM search implemented in Perl was used to compare each of the *P. damicornis *contigs to the conserved protein domains housed at Pfam; ftp://ftp.sanger.ac.uk/pub/databases/Pfam/Tools/OldPfamScan/pfam_scan.pl). The "Pfam" table links each PFID to the name and description of the relevant protein domain. BLASTX output is summarized in the "Blast" table. Here, each CID is associated with up to five protein sequences in NCBI that produced significant BLASTX hits to the relevant *P. damicornis *contig. Various metrics from the BLAST hits are linked to each protein identifier (PID) including the BLAST alignment length, the bit score, the expect score, and the number of identical amino acids. Through an "Annotation" table, the PIDs are cross-referenced with gene ontology IDs (GOID). The "Ontology" table links each GOID to the name and description of the relevant gene ontology term. The "Species" table relates each PID to the species from which the corresponding protein sequence was derived and indicates whether that species is a known symbiont of *P. damicornis*. The "Accession" table cross-references each PID with the corresponding mRNA ID (MID) if this information is available in NCBI. The "mRNA" table links these MIDs to the corresponding mRNA name and nucleotide sequence. Through the "Tapp" table, each PID may be cross-referenced to 0, 1, or more biochemical pathways housed in the KEGG database. The "Kegg" table associates each Kegg ID (KID) with the name and definition of the corresponding biochemical pathway.

The database can be accessed via an HTML interface at http://www.PocilloporaBase.org. Users can query the database via any one of ten different search terms (described in detail in the Utility and Discussion section). Database queries are generated using the Python programming language, version 2.6.5, and the PyGreSQL DB-API module. Web pages summarizing the search results are produced as needed via Python scripts that generate HTML output. The database is hosted on a computer running Ubuntu Linux 10.04.2 using the Apache HTTP Server (version 2.2.14).

### Adult coral RNA extraction, and sequencing

**Figure 2:**
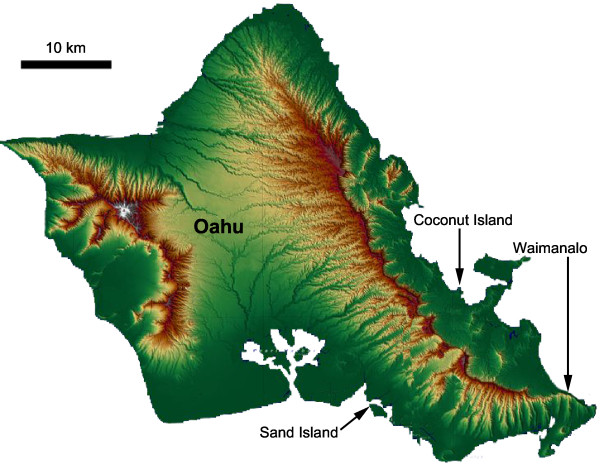
**Collection sites for adult *P. damicornis***. Adult *P. damicornis *was collected from three geographically isolated populations in Oahu, Hawaii: Coconut Island, a site found on the north eastern corner of Oahu; Sand Island, a site found on the southern part of Oahu, just west of Honolulu; and Waimanalo a site found on the eastern side of Oahu.

Adult *P. damicornis *colonies were collected from three geographically isolated populations in Oahu, Hawaii (Figure 2). Coconut Island, on the northeastern corner of Oahu, is considered to be "recovering" from significant human impacts [23]. Sand Island, off the southern coast of Oahu, just west of Honolulu, abuts a heavily industrialized area that houses the chief sewage treatment plant for all of metropolitan Honolulu [24]. Relative to these two sites, Waimanalo, on the eastern side of Oahu is relatively un-impacted by human activity. Three to four individual colonies were collected from each site.

**Figure 3:**
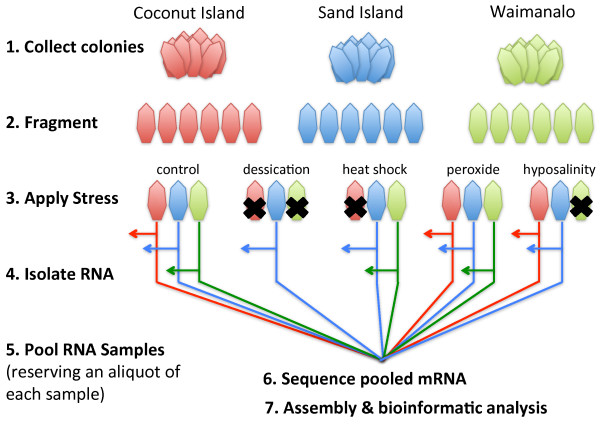
**Summary of *P. damicornis *transcriptome sequencing workflow**. Healthy corals were collected from three different sites in Oahu, Hawaii and exposed to five environmentally relevant stressors (heat, desiccation, peroxide, and hypo-saline conditions) or control conditions. RNA was extracted from healthy populations and stressed populations and pooled together. (RNA isolated from four colony/treatments was not of sufficient quality (black x's) to be used in library preparation, so it was not subjected to further processing.) Library construction, sequencing, and assembly were performed by Agencourt Genomic Services (now Beckman Coulter Genomics); sequencing was performed on the 454 sequencing platform.

Upon collection, each colony was fragmented into nubbins, and the nubbins were kept in an outdoor seawater table for two weeks before being subjected to a range of biologically relevant stressors administered in a controlled laboratory setting. The stressors included desiccation (four hours out of water), hypo-saline shock (two hours in fresh water), heat shock (50°C for 1 hour), and peroxide exposure (2 hours in sea water supplemented with 10% peroxide). Total RNA from stressed and control nubbins was extracted using Trizol [25]. To produce the reference transcriptome described here, aliquots of all the individual RNA samples were pooled prior to sequencing. The pooled RNA sample was then shipped to Beckman Coulter for preparation of a non-normalized library and sequencing using the 454 sequencing technology [26,27] (Figure 3). The remainder of each individual RNA sample is being sequenced separately on the Illumina platform so that we might identify transcriptional changes associated with particular stressors (unpublished data). Three RNA samples (one from each location) were submitted to the Ocean Genome Legacy's Ocean Genome Resource database (accession numbers: S06518, S06519, and S06520).

### Assembly, Identification of contigs, and Pfam domain searches

After sequencing, short reads (< 40 nucleotides) and low quality reads that did not overlap with other sequencing reads were discarded, and the remaining 955, 910 sequencing reads were assembled at Beckman Coulter using MIRA3 [28]. High quality singletons were included with the contigs in all subsequent analyses. Contig sequences were blasted against the adaptor sequences used for both the library preparation and the sequencing to insure that none of the contig sequences were contaminated with adaptors. Adaptor sequences were trimmed, and the assembled contigs were used to sequentially query the NCBI non-redundant protein database using BLASTX with an E-value cut-off of 0.001. The top five gene hits were assigned to each contig. All five of the top hits usually agreed on gene ontology and taxonomy, but where they disagreed, we associated multiple GO terms and multiple possible taxonomic affinities with a given contig. In order to identify conserved protein domains, all six open reading frames were blasted against the protein domain database at Pfam [29]. Hits were retained only if they had an e-value cut off lower than 0.001.

### GO term and KEGG analysis

Using the top five hits from the BLASTX search described above, each contig was assigned a list of associated protein GI numbers. GI numbers were converted to Entrez Gene IDs using the gene2accession conversion file from NCBI. Gene2go was then used to obtain relevant GO annotation for the five top BLASTX hits to each *P. damicornis *contig, and the GO term(s) were then associated with their respective contig. Protein GI numbers were cross-referenced to species-specific KEGG pathways [30]. Using KEGG's lists of annotated plants and animals, these pathways were organized into corresponding lists, and generalized KEGG pathway Ids were obtained. KEGG pathway analysis was then performed, and individual contigs were then mapped to different biochemical pathways using IPath [22]. The top 5 hits were chosen to increase the probability of finding a hit that would allow pairing each contig with its corresponding GO category and with the existent KEGG data. Using photosynthesis as an example, the top hits matched to *Symbiodinium *sequences, not plants. Because *Symbiodinium *is not represented within the KEGG database, the contigs did not appear to represent enzymes involved in photosynthesis, when, in fact, they did.

## Utility and Discussion

### Sequencing yield

Sequencing yielded 1, 116, 551 raw reads with an average length of 379 nucleotides (range: 29-2, 025 nt; SD = 152 nt). Reads less than 40 nucleotides in length and low quality reads that did not overlap with other reads were discarded. The remaining 955, 105 reads were assembled into 70, 786 contigs with an average length of 836 nt (range: 40-10, 512 nt; SD = 464 nt; Additional File 2; 3). These data are compared with other published scleractinian transcriptomic data sets in Table 1.

### Taxonomic affinity of the sequences

**Figure 4:**
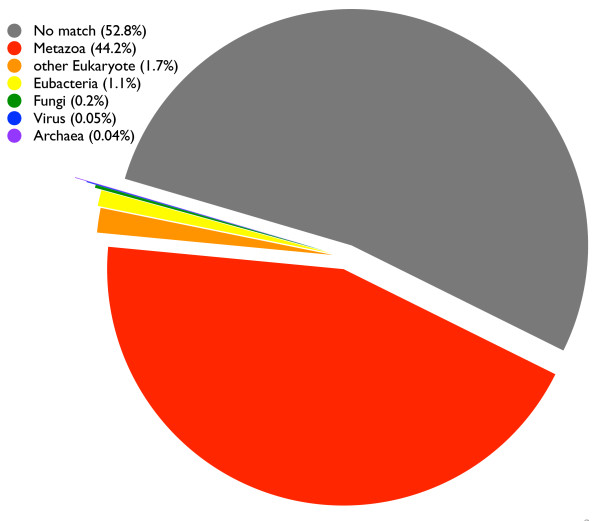
**Taxonomic affinities of contig sequences based on BLAST**. Overall, 52.9% (or 37, 423) of the contigs matched sequences from NCBI with an E-value cutoff of 0.001. Of the 37, 423 hits, 31, 271 appeared metazoan, 139 fungal, 36 viral, 764 eubacterial, 26 archaeal, and 1187 hits as "other" eukaryote.

We used the top hit in a BLASTX [PMID: 2231712] search to characterize each of the assembled contigs according to its apparent taxonomic affinity. Overall, 47.2% (or 33, 423) of the contigs matched sequences housed at NCBI with an E-value cutoff of 0.001. The other 37, 363 contigs did not match sequences at NCBI with an E-value of ≤.001 and were excluded from subsequent analyses. Of the 33, 423 hits, 31, 271 appeared metazoan, 139 fungal, 36 viral, 764 eubacterial, and 26 archaeal (Figure 4). We classified 1187 hits as "other eukaryote;" when these other eukaryotic hits were parsed further, 142 matched a sequence from *Symbiodinium*, the genus of unicellular algae that are intracellular endosymbionts of hermatypic corals (Table 2). For a complete breakdown of BLAST matches to other eukaryotes including dinoflagellates, see Additional Files 4, 5.

**Table 2:** Summary of *Symbiodinium *blast hits

Gene/Protein Name	NCBI ID
Actin	87116473
	87116475
Bacl-2	119710160
Dna J-like protein 1	75858825
Dna J-like protein 2	75858827
Dna J-like protein 3	75858829
Glyceraldehyde-3-phosphate dehydrogenase	32454981
	35210444
	35210448
	35210454
Heat shock protein 70	75858823
Heat shock protein 90	75858821
Peridinin chlorophyll-a binding protein apoprotein precursor	23986591
	23986608
	23986610
	23986617
	23986634
	23986641
	1709613
	23986384
	23986401
	23986430
	23986551
Phosphoglycolate phosphatase	197091190
Polyubiquitin	75858833
Ribulose bisphosphate carboxylase	75282236
Ubiquitin ligase 2	75858845
Ubiquitin-specific protease 1	75858847
Unknown	134035981
	134035977

The taxonomy of *Pocillopora *is currently regarded as tenuous, *e.g*., some 16 species have been defined on the basis of morphological features, but cladistic groupings defined by molecular sequence data are not always congruent with these morphologically defined taxa (*e.g*., [31]). We used reciprocal blast searches to investigate whether the sequences we generated in this study most closely resemble *P. damicornis *sequences in NCBI, rather than sequences from other closely related corals, including other *Pocillopora *species. We obtained 400 "*P. damicornis*" ESTs obtained from NCBI, and we used these to query the contigs housed at PocilloporaBase (blastn with an E-value cut-off of 0.001). Because a substantial fraction of the *P. damicornis *sequences currently housed at NCBI represent multiple copies of the same gene (generated in population genetics studies,) many of them matched to the same contig in PocilloporaBase. Overall, the 400 sequences from NCBI matched to 21 unique contigs at PocilloporaBase. We blasted these 21 contigs back against all nucleotide sequences at NCBI classified as scleractinian (Search "Scleractinia[Organism]"). The results are provided in Additional File 6. In 6 of 21 instances, the only match in the database was to a *P. damicornis *sequence. In 12 instances, there were matches to other corals in addition to *P. damicornis*, but the highest degree of sequence identity was to a sequence from *P. damicornis*. In one instance, a contig from PocilloporaBase exhibited equal percent identity to sequences from *P. damicornis *and *P. meandrina*. Finally, there were two contigs that exhibited a slightly higher resemblance to a sequence from a coral other than *P. damicornis*. On balance, these results clearly suggest that the "*P. damicornis*" populations sampled in this study exhibit greater sequence similarity to the *P. damicornis *sequences housed at NCBI than to any other coral species represented in that database. However, the taxonomic uncertainty of greatest concern pertains specifically to the genus *Pocillopora*. Here, the results are less decisive. Only seven of the twenty-one contig sequences we blasted against the NCBI database produced hits to sequences from *P. damicornis *and another *Pocillopora *species. In five of these seven instances, the top hit was to a *P. damicornis *sequence, but one contig matches better to *P. molokensis *and another matches equally well to *P. damicornis *and *P. meandrina*.

### Gene Ontology

**Figure 5:**
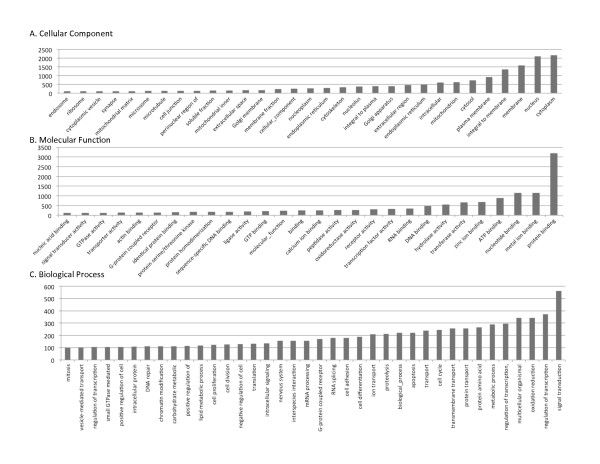
**Gene ontology terms associated with *P. damicornis *sequences**. The number of *P. damicornis *contigs associated with each GO term is shown for Cellular Compartment, Molecular Function, and Biological Function. Only GO terms associated with 100 or more *P. damicornis *sequences are shown.

One or more GO annotation terms could be associated with 23, 202 of the 70, 786 contigs (see Construction and content); 7, 084 contigs had a unique GO annotation. All of the GO terms that were attributed to at least 100 contigs are summarized in Figure 5 (for a complete listing, see Additional File 7).

### KEGG Pathway Analysis

The contigs were subjected to the KEGG Pathway analysis, for human and plant separately [30]. Based on this analysis, the *P. damicornis *sequences were mapped to metabolic pathways on the interactive tree of life [32]. Components of most metabolic pathways were identified, including photosynthesis (KEGG id: map00195 and 00196) and lipid metabolism. However, some pathways were largely or completely absent from the *P. damicornis *contigs, including aminosugars metabolism (00530), lipopolysaccharide biosynthesis (00540), peptidoglycan biosynthesis (00550), glycosphingolipid biosynthesis (00602), methane metabolism (00680), androgen and estrogen metabolism (00150), and biodegradation of most xenobiotics (Additional Files 8, 9).

### PocilloporaBase: capabilities and functions

PocilloporaBase http://www.PocilloporaBase.org was modeled after StellaBase, a genomic and transcriptomic database for the starlet sea anemone, *Nematostella vectensis *[33,34]. Both species-specific databases were designed to integrate with CnidBase [35], a phylum-wide database meant to facilitate cross-species comparisons among cnidarians. All of the raw sequencing reads generated in this study as well as the assembled contigs can be downloaded there. At present, unlike StellaBase, PocilloporaBase houses only transcriptome data and no genomic sequence data. This repository will grow as future mRNA and genomic sequencing projects generate additional data for *Pocillopora damicornis*.

**Figure 6:**
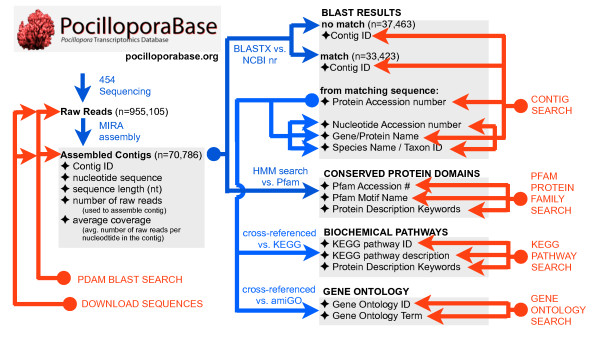
**Functionality of PocilloporaBase**. PocilloporaBase houses the raw sequencing reads generated in this study as well as the assembled contigs that were generated from the sequencing reads and the output from a number of bioinformatic analyses performed on the contigs. The contigs were used to search the non-redundant database at NCBI using BLASTX. The contigs were also used to conduct an HMM search of the Pfam database to identify conserved protein motifs. The proteins producing significant matches to *P. damicornis *contigs in the BLAST search were cross-referenced with biochemical pathways at KEGG and with gene ontology terms at amiGO. Users can search the *Pocillopora *contigs based on features of the protein they matched in the BLAST search or conserved Pfam protein domains they appear to encode. Users can also search either the contigs or the raw reads using BLAST, and all of the sequencing reads and contigs can be downloaded from the site. Steps taken to populate the database are represented by blue arrows. Actions available to the user are represented by red arrows.

The assembled contigs housed at PocilloporaBase can be searched using several different modalities (Figure 6). The Contig Search allows you to query sequences using NCBI Protein Accession Number (*e.g*.: 74000907), Nucleotide Accession Number (*e.g*.: 33340018), Gene/Protein Name (e.g., 'hemoglobin'), or Species Name/Taxon ID (e.g.: 'otolemur' or 45351). The option of searching the data by organism is critical, since corals can be considered a holobiont consisting of coral host, symbiotic algae (*Symbiodinium*), bacteria and fungi.

The Gene Ontology Search allows you to query sequences using either GO id or GO description terms [36]. Each successful query returns a table that contains the Protein ID and Protein Name, as well as GO id, GO description, and GO type. The Protein ID links to the protein's entry at NCBI. The Protein Name links to a Protein Lookup on PocilloporaBase, which returns a list of *P. damicornis *contigs that generated significant BLAST hits to the protein in question. The GO id links to the corresponding gene ontology page at the amiGO database. Clicking on the GO description link performs a more specific GO lookup if any gene ontology terms are children of the parent term used to conduct the original search. If too many results are returned, searches can be restricted to one of the three principal gene ontology types: biological process, cellular component, or molecular function. If the user is more interested in the number rather than the identity of the genes in the database that map to a particular gene ontology term, the "Counts only" box can be checked.

The KEGG Pathway Search allows you to query sequences using a KEGG ID number (e.g., hsa00010) or KEGG Pathway Description (*e.g*., Glycolysis/Gluconeogenesis). Each successful query returns a table with individual contigs identified by their Contig ID, the E-value of their match to a protein in the KEGG database, the KEGG Organism ID, homologous protein name and protein accession ID.

The presence of conserved protein motifs in one or more transcripts can be investigated by searching the data for matches to the conserved protein motifs housed at Pfam. The Pfam Protein Family Classification search allows you to query sequences by Pfam Accession number (*e.g*., PF00006), Motif Name (*e.g*.: PAX, actin, DNA_methylase) or Protein Description key words. If there is a match to a conserved protein motif at Pfam, the search returns a table of *Pocillopora damicornis *contigs encoding that motif sorted by E-value.

It is also possible to search for matches to a query sequence using the complete set of BLAST options. BLAST searches return contig id, the sequence for that contig, as well as the NCBI gene ID, gene name, and the gene sequence for any gene sequence found to match the original blast query. A gene search page allows for quick retrieval of gene and species information in the database.

## Conclusions

We used the 454 sequencing platform to generate a reference transcriptome for the cauliflower coral, *P. damicornis*. A taxonomic analysis of the sequence data indicates that we have captured some of the diversity of the coral holobiont, as many of the sequences appear to be derived from non-metazoan taxa including bacteria, fungi, viruses, and unicellular algae of the genus *Symbiodinium*. The data have been organized into a publicly available relational database that will be updated and expanded as new *P. damicornis *sequencing data become available.

## Availability and requirements

This database can be accessed using a web browser at http://www.pocilloporabase.org.

## Authors' contributions

NTK collected the animals, subjected the nubbins to stressors, isolated the RNA, and drafted the manuscript. BG, TL, JRP, SG, YX, and JAM performed bioinformatics analyses on the data, constructed the database, and built the internet interface for PocilloporaBase. LK contributed to the conception of the project and drafting of the manuscript. JRF contributed to the conception of the project, data analysis and interpretation, production of the figures, and drafting of the manuscript. All authors read and approved the final manuscript.

## Supplementary Material

Additional file 1 **A diagram depicting all of PocilloporaBase's tables and entity relationships as well as definitions for all fields**. Click here for file

Additional file 2 **A frequency histogram of the lengths of the assembled contigs in nucleotides**. The number of contigs was summed for every 100-nucleotide increment in length (*e.g*., 0-99 nt, 100-199 nt; *etc*.). Click here for file

Additional file 3 **A compressed text file containing all 70, 786 contigs reported in the manuscript**. Click here for file

Additional file 4 **A pie chart summarizing the taxonomic affinities of non-metazoan, non-fungal, eukaryotic hits returned by BLAST searches**. Click here for file

Additional file 5 **A pie chart summarizing the taxonomic affinities of top hits to sequences from Alveolates (including dinoflagellates) returned by BLAST searches**. Click here for file

Additional file 6 **Blast results from using *P. damicornis *contigs to query NCBI for scleractinian sequences**. Click here for file

Additional file 7 **A complete listing of Gene Ontology terms and BLAST matches for all sequences**. Click here for file

Additional file 8 **A diagram depicting KEGG pathway elements and whether they were found or not found among the *P. damicornis *contigs**. Click here for file

Additional file 9 **A compressed folder containing an interactive (iPath) version of Additional File **7, **which shows the metabolic pathways present in *P. damicornis *as inferred by BLASTx matches to human or plant genes with known metabolic functions**. Click here for file
