# Supplementary material for: Production of a reference transcriptome and transcriptomic database (PocilloporaBase) for the cauliflower coral, Pocillopora damicornis
Source: BMC Genomics. 2011 Nov 29;12:585. doi: 10.1186/1471-2164-12-585 (PMC3339375; doi:10.1186/1471-2164-12-585)
Supplement: Additional file 1 — A diagram depicting all of PocilloporaBase's tables and entity relationships as well as definitions for all fields. [file 1471-2164-12-585-S1.PDF]

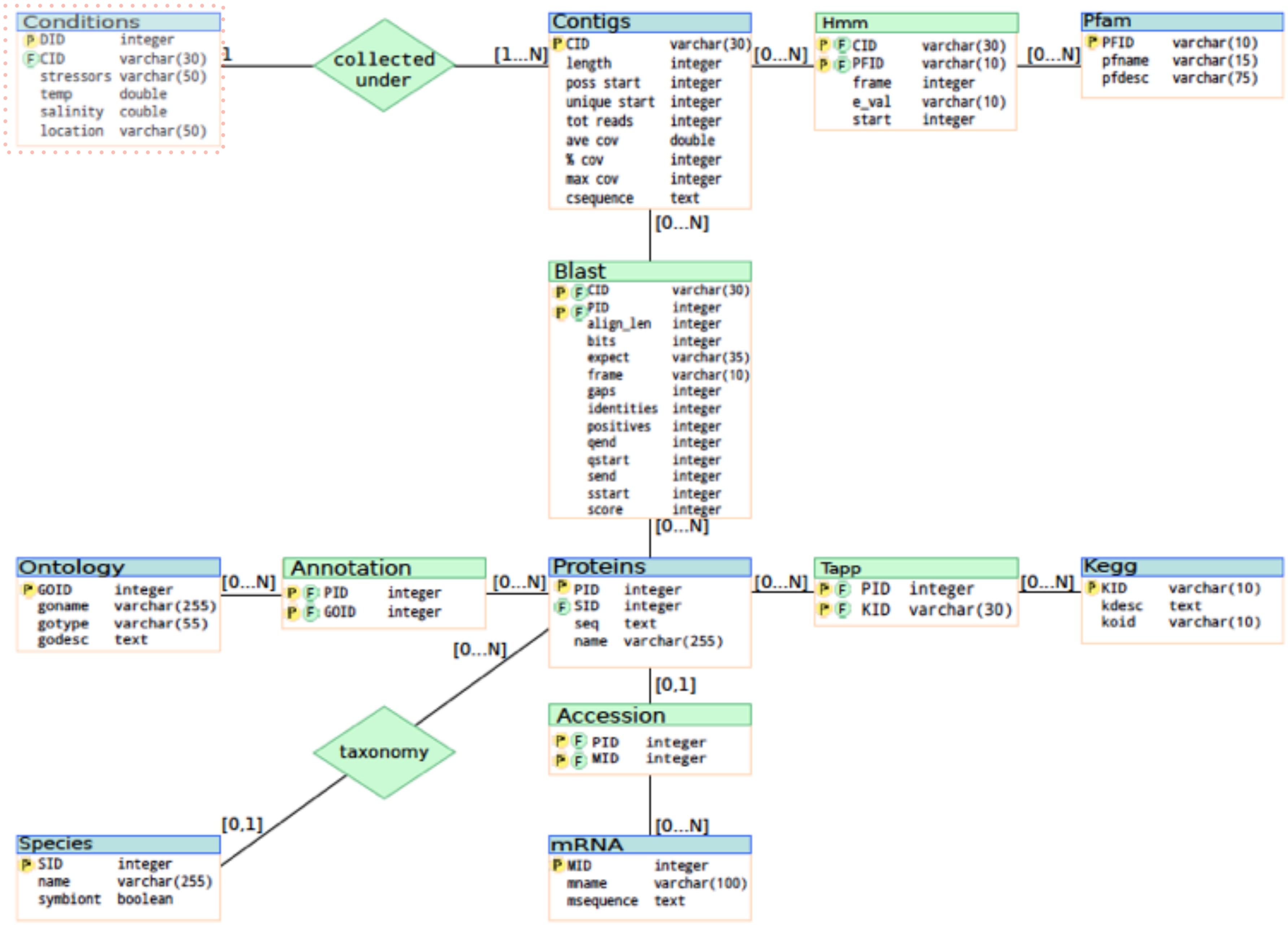

Conditions
P DID integer
F CID varchar(30)
stressors varchar(50)
temp double
salinity couble
location varchar(50)
1
collected under
[1...N]
Contigs
P CID varchar(30)
length integer
poss start integer
unique start integer
tot reads integer
ave cov double
% cov integer
max cov integer
csequence text
[0...N]
Hmm
P F CID varchar(30)
P F PFID varchar(10)
frame integer
e_val varchar(10)
start integer
[0...N]
Pfam
P PFID varchar(10)
pfname varchar(15)
pfdesc varchar(75)
[0...N]
Blast
P F CID varchar(30)
P F PID integer
align_len integer
bits integer
expect varchar(35)
frame varchar(10)
gaps integer
identities integer
positives integer
qend integer
qstart integer
send integer
sstart integer
score integer
[0...N]
Ontology
P GOID integer
goname varchar(255)
gotype varchar(55)
godesc text
[0...N]
Annotation
P F PID integer
P F GOID integer
[0...N]
Proteins
P PID integer
F SID integer
seq text
name varchar(255)
[0...N]
Tapp
P F PID integer
P F KID varchar(30)
[0...N]
Kegg
P KID varchar(10)
kdesc text
koid varchar(10)
[0...N]
taxonomy
[0,1]
Species
P SID integer
name varchar(255)
symbiont boolean
[0,1]
Accession
P F PID integer
P F MID integer
[0...N]
mRNA
P MID integer
mname varchar(100)
msequence text

Structure Of PocilloporaBase.
Each table in the database is represented by a box. Entities are represented by boxes with blue headings. Relations are represented with green headings when a table is present in the database, or by a green diamond when a database table was not necessary. “P” indicates the primary key of a table, and “F” indicates a foreign key. Lines are drawn between tables to show connections, and numbers along the connections indicate cardinality between tables. In each table, the left column indicates the variable name, and the right column indicates the variable type. The Conditions table is ghosted because this table does not yet have any entries in it. The Conditions table will be populated as RNA sequence data derived from animals obtained from different geographic locations or subjected to different environmental conditions are deposited in the database
