# Supplementary figures and images for: Production of a reference transcriptome and transcriptomic database (PocilloporaBase) for the cauliflower coral, Pocillopora damicornis
Source: BMC Genomics. 2011 Nov 29;12:585. doi: 10.1186/1471-2164-12-585 (PMC3339375; doi:10.1186/1471-2164-12-585)

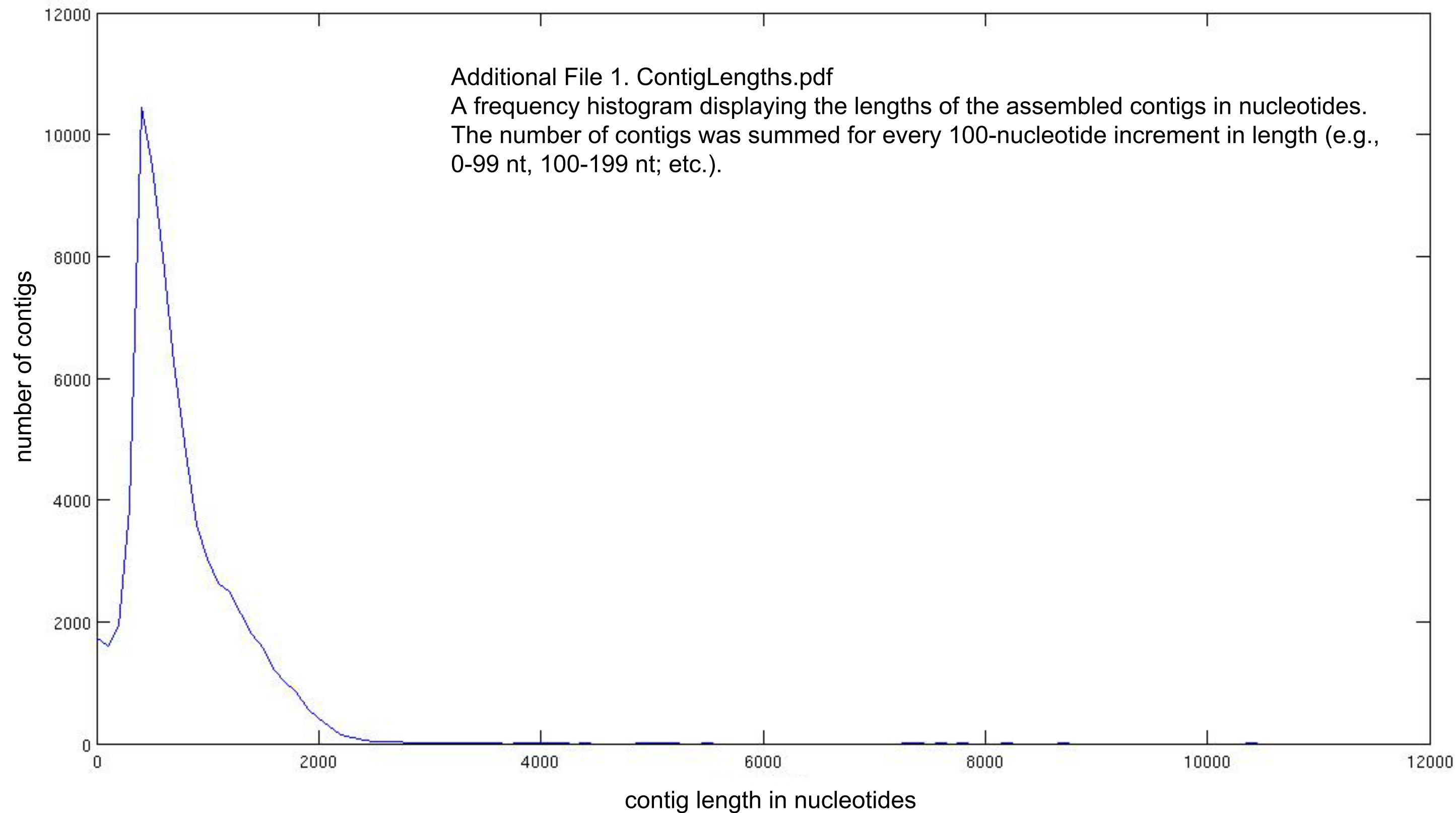

Supplement: Additional file 2 — A frequency histogram of the lengths of the assembled contigs in nucleotides. The number of contigs was summed for every 100-nucleotide increment in length (e.g., 0-99 nt, 100-199 nt; etc.). [file 1471-2164-12-585-S2.PDF]

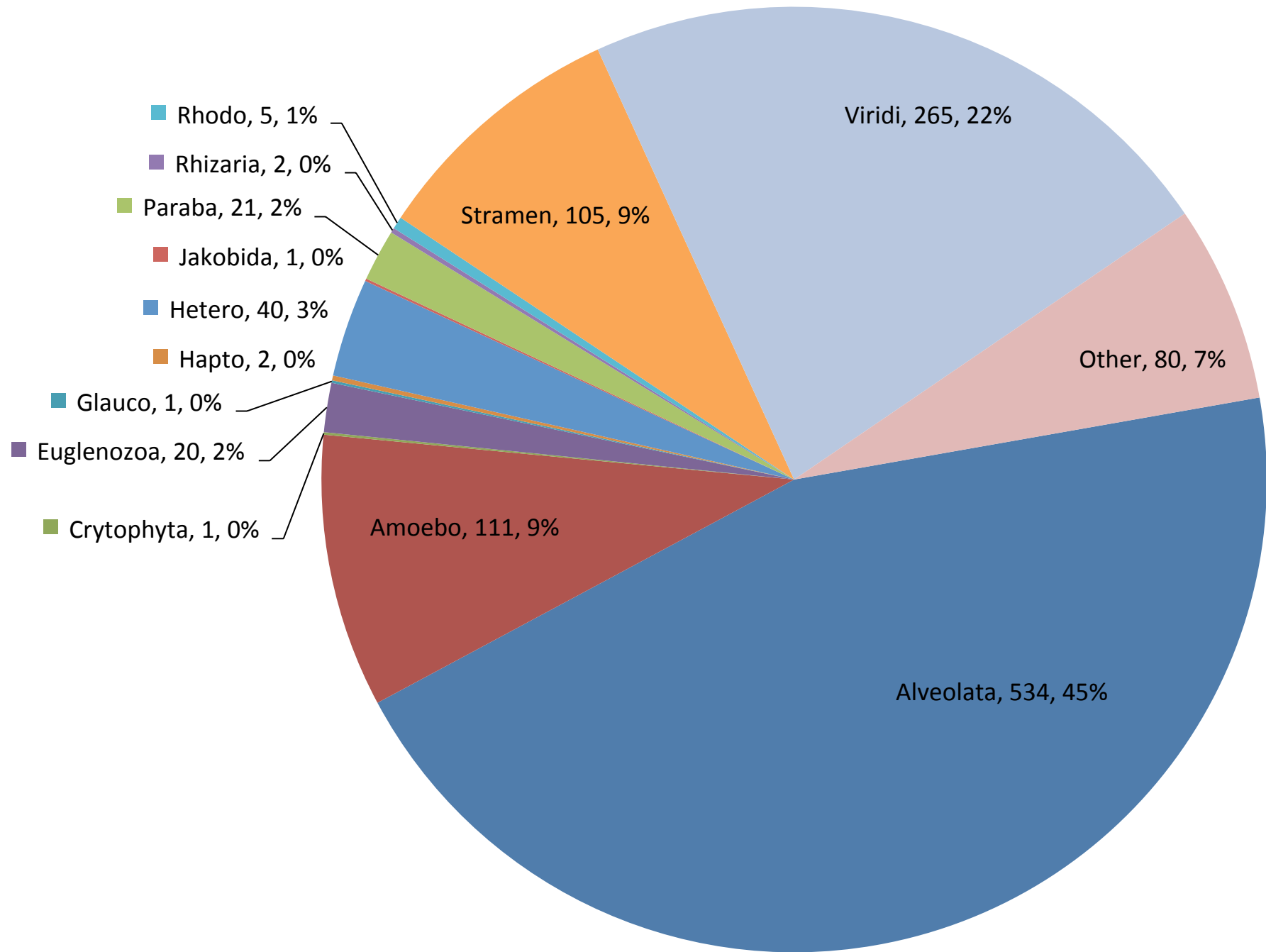

Supplement: Additional file 4 — A pie chart summarizing the taxonomic affinities of non-metazoan, non-fungal, eukaryotic hits returned by BLAST searches. [file 1471-2164-12-585-S4.PDF]

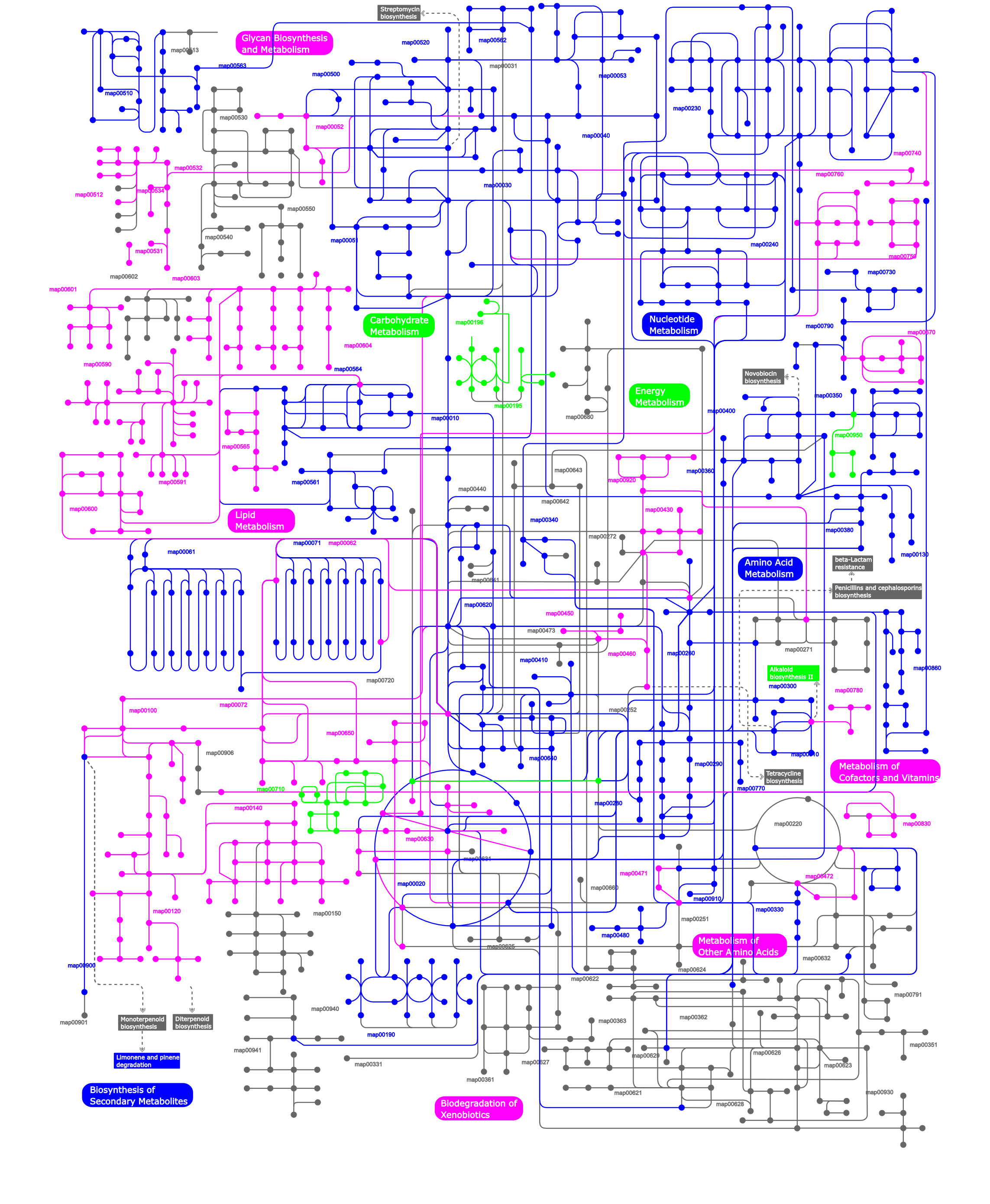

Supplement: Additional file 9 — A compressed folder containing an interactive (iPath) version of Additional File 7, which shows the metabolic pathways present in P. damicornis as inferred by BLASTx matches to human or plant genes with known metabolic functions. [file 1471-2164-12-585-S9.ZIP › rekeggfigure/kegg_top5.png]
