# Supplementary material for: Production of a reference transcriptome and transcriptomic database (PocilloporaBase) for the cauliflower coral, Pocillopora damicornis
Source: BMC Genomics. 2011 Nov 29;12:585. doi: 10.1186/1471-2164-12-585 (PMC3339375; doi:10.1186/1471-2164-12-585)
Supplement: Additional file 5 — A pie chart summarizing the taxonomic affinities of top hits to sequences from Alveolates (including dinoflagellates) returned by BLAST searches. [file 1471-2164-12-585-S5.PDF]

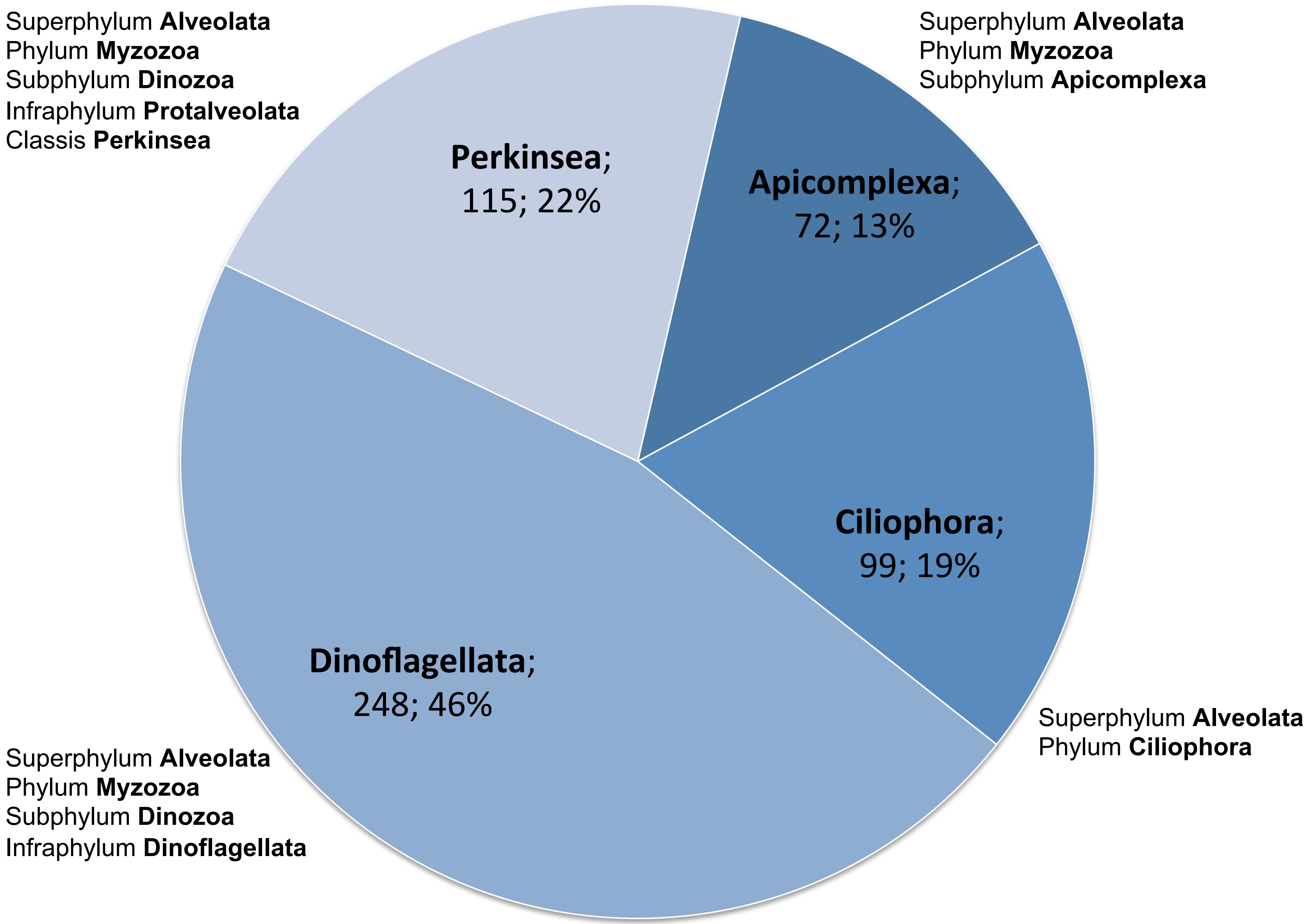
Superphylum **Alveolata**
Phylum **Myzozoa**
Subphylum **Dinozoa**
Infraphylum **Protalveolata**
Classis **Perkinsea**
Superphylum **Alveolata**
Phylum **Myzozoa**
Subphylum **Apicomplexa**
Perkinsea; 115; 22%
Apicomplexa; 72; 13%
Ciliophora; 99; 19%
Dinoflagellata; 248; 46%
Superphylum **Alveolata**
Phylum **Ciliophora**
Superphylum **Alveolata**
Phylum **Myzozoa**
Subphylum **Dinozoa**
Infraphylum **Dinoflagellata**
