# Supplementary material for: Production of a reference transcriptome and transcriptomic database (PocilloporaBase) for the cauliflower coral, Pocillopora damicornis
Source: BMC Genomics. 2011 Nov 29;12:585. doi: 10.1186/1471-2164-12-585 (PMC3339375; doi:10.1186/1471-2164-12-585)
Supplement: Additional file 6 — Blast results from using P. damicornis contigs to query NCBI for scleractinian sequences. [file 1471-2164-12-585-S6.PDF]

Contig id	Scleractinia g	taxon id	Taxon name	percent iden	length alignment	mismatches	gaps	query start	query end	subject start	subject end	E-value	bits
bu_91849.1_c4	gi|16797883	45264	Acropora millepora	82.03	1096	171	26	694	1776	1169	87	0	909
bu_91849.1_c4	gi|15443469	46731	Pocillopora damicornis	99.39	493	2	1	1291	1783	522	31	0	893
bu_91849.1_c4	gi|22281981	45264	Acropora millepora	82.81	983	147	22	694	1665	972	1	0	859
bu_91849.1_c1947	gi|26040090	46731	Pocillopora damicornis	97.74	266	4	2	953	1217	265	1	9.00E-128	457
bu_91849.1_c3553	gi|11935226	46731	Pocillopora damicornis	98.14	161	0	3	996	1153	1	161	7.00E-74	278
bu_91849.1_c3553	gi|22278209	45264	Acropora millepora	95.35	43	2	0	104	146	456	414	4.00E-11	69.4
bu_91849.1_c6421	gi|29500368	51051	Acanthastrea echinata	98.81	675	6	2	60	733	21	694	0	1203
bu_91849.1_c6421	gi|29500373	227230	Pavona decussata	98.81	675	6	2	60	733	21	694	0	1203
bu_91849.1_c6421	gi|29500370	46731	Pocillopora damicornis	98.52	675	8	2	60	733	21	694	0	1195
bu_91849.1_c6421	gi|29500371	46731	Pocillopora damicornis	98.37	676	7	4	60	733	21	694	0	1186
bu_91849.1_c6421	gi|29500369	46731	Pocillopora damicornis	98.22	676	8	4	60	733	21	694	0	1181
bu_91849.1_c14445	gi|89994687	46731	Pocillopora damicornis	99.21	1650	12	1	16	1665	17	1665	0	2974
bu_91849.1_c14445	gi|22254812	45264	Acropora millepora	81.59	201	31	5	811	1008	40	237	9.00E-39	161
bu_91849.1_c14921	gi|17029720	46731	Pocillopora damicornis	95.31	213	7	3	189	399	473	262	2.00E-91	335
bu_91849.1_c14921	gi|17029721	46731	Pocillopora damicornis	95.28	212	9	1	189	399	473	262	2.00E-91	335
bu_91849.1_c14921	gi|17029725	512010	Pocillopora ligulata	95.28	212	9	1	189	399	491	280	2.00E-91	335
bu_91849.1_c14921	gi|17029721	46731	Pocillopora damicornis	94.81	212	10	1	189	399	491	280	8.00E-90	329
bu_91849.1_c14921	gi|17029721	46731	Pocillopora damicornis	94.81	212	10	1	189	399	489	278	8.00E-90	329
bu_91849.1_c14921	gi|17029722	46731	Pocillopora damicornis	94.81	212	10	1	189	399	491	280	8.00E-90	329
bu_91849.1_c14921	gi|17029723	46731	Pocillopora damicornis	94.81	212	10	1	189	399	491	280	8.00E-90	329
bu_91849.1_c14921	gi|17029725	512010	Pocillopora ligulata	94.81	212	10	1	189	399	491	280	8.00E-90	329
bu_91849.1_c14921	gi|17029725	512010	Pocillopora ligulata	94.81	212	10	1	189	399	489	278	8.00E-90	329
bu_91849.1_c14921	gi|17029725	512010	Pocillopora ligulata	94.81	212	10	1	189	399	491	280	8.00E-90	329
bu_91849.1_c14921	gi|17029725	512010	Pocillopora ligulata	94.81	212	10	1	189	399	491	280	8.00E-90	329
bu_91849.1_c14921	gi|17029725	512010	Pocillopora ligulata	94.81	212	10	1	189	399	491	280	8.00E-90	329
bu_91849.1_c14921	gi|17029725	512010	Pocillopora ligulata	94.81	212	10	1	189	399	491	280	8.00E-90	329
bu_91849.1_c14921	gi|17029725	512010	Pocillopora ligulata	94.81	212	10	1	189	399	491	280	8.00E-90	329
bu_91849.1_c14921	gi|17029726	512010	Pocillopora ligulata	94.81	212	10	1	189	399	491	280	8.00E-90	329
bu_91849.1_c14921	gi|17029726	512010	Pocillopora ligulata	94.81	212	10	1	189	399	491	280	8.00E-90	329
bu_91849.1_c14921	gi|17029726	512010	Pocillopora ligulata	94.81	212	10	1	189	399	491	280	8.00E-90	329
bu_91849.1_c14921	gi|17029726	512011	Pocillopora molokensis	94.81	212	10	1	189	399	491	280	8.00E-90	329
bu_91849.1_c14921	gi|17029726	512011	Pocillopora molokensis	94.81	212	10	1	189	399	491	280	8.00E-90	329
bu_91849.1_c14921	gi|17029727	512011	Pocillopora molokensis	94.81	212	10	1	189	399	491	280	8.00E-90	329
bu_91849.1_c14921	gi|17029727	512011	Pocillopora molokensis	94.81	212	10	1	189	399	475	264	8.00E-90	329
bu_91849.1_c14921	gi|17029725	512010	Pocillopora ligulata	94.34	212	11	1	189	399	491	280	4.00E-88	324
bu_91849.1_c14921	gi|17029726	367768	Pocillopora eydouxi	93.87	212	11	2	189	399	490	280	2.00E-86	318
bu_91849.1_lrc17272	gi|58865329	46731	Pocillopora damicornis	98.18	1597	17	11	12	1607	924	2509	0	2789
bu_91849.1_lrc17272	gi|16963506	46704	Montipora capitata	87.13	272	31	4	402	671	4	273	4.00E-82	305
bu_91849.1_c18936	gi|59275992	46731	Pocillopora damicornis	96.93	293	9	0	79	371	7	299	2.00E-138	492
bu_91849.1_c19034	gi|5441274|	86588	Phyllangia mouchezii	96.01	1805	55	15	6	1806	6	1797	0	2918
bu_91849.1_c19034	gi|5441271|	46714	Lobactis scutaria	95.84	1805	58	14	6	1806	6	1797	0	2902
bu_91849.1_c19034	gi|5441272|	44301	Enallopsammia rostrata	95.84	1805	58	14	6	1806	6	1797	0	2902
bu_91849.1_c19034	gi|5931759|	46700	Tubastraea coccinea	95.75	1812	50	25	6	1809	5	1797	0	2894
bu_91849.1_c19034	gi|12247086	48500	Montastraea annularis	95.79	1804	59	14	6	1805	1	1791	0	2894
bu_91849.1_c19034	gi|15208495	48499	Montastraea franksi	95.62	1805	62	14	6	1806	26	1817	0	2880
bu_91849.1_c19034	gi|5441270|	46726	Pavona varians	95.51	1804	66	12	6	1806	6	1797	0	2868
bu_91849.1_c19034	gi|5441273|	86586	Ceratotrochus magnaghii	95.11	1820	59	26	6	1806	6	1814	0	2841
bu_91849.1_c19034	gi|2370433|	58808	Rhizopsammia minuta	95.14	1812	62	24	6	1809	5	1798	0	2835
bu_91849.1_c19034	gi|14700052	165419	Astrangia poculata	95.32	1794	65	16	6	1796	6	1783	0	2830
bu_91849.1_c19034	gi|5931758|	91457	Javania insignis	94.78	1821	58	31	6	1809	5	1805	0	2802

bu_91849.1_c19034	gi|15778332	46753	Fungiacyathus marenzelleri	94.67	1820	66	23	6	1806	6	1813	0	2795
bu_91849.1_c19034	gi|61697101	123771	Madracis mirabilis	96.07	1703	52	13	49	1748	3	1693	0	2760
bu_91849.1_c19034	gi|2370579|	46700	Tubastraea coccinea	93.61	1814	85	28	6	1809	5	1797	0	2678
bu_91849.1_c19034	gi|16294581	50429	Stylophora pistillata	97.42	814	17	4	13	825	6	816	0	1386
bu_91849.1_c19034	gi|61697100	123769	Madracis decactis	96.35	794	21	7	106	896	1	789	0	1299
bu_91849.1_c19034	gi|19713096	46731	Pocillopora damicornis	99.85	666	1	0	487	1152	1	666	0	1225
bu_91849.1_c19034	gi|1518533|	47068	Balanophyllia elegans	94.23	797	35	9	1009	1803	1	788	0	1206
bu_91849.1_c19034	gi|34555710	244863	Tubastraea sp. SR-2003	95.91	684	19	3	336	1010	1	684	0	1105
bu_91849.1_c19034	gi|34555711	242744	Cynarina lacrymalis	95.91	684	19	3	336	1010	1	684	0	1099
bu_91849.1_c19034	gi|34555709	75303	Heliofungia actiniformis	95.61	684	21	9	335	1010	2	684	0	1088
bu_91849.1_c19034	gi|58334050	174260	Lophelia pertusa	93.52	725	36	9	1023	1744	12	728	0	1068
bu_91849.1_c19034	gi|34555707	46748	Catalaphyllia jardinei	93	686	35	10	336	1010	1	684	0	989
bu_91849.1_c19034	gi|34555705	244860	Plerogyra sp. SR-2003	92.85	685	36	10	336	1010	1	682	0	981
bu_91849.1_c19034	gi|34555704	244858	Lobophyllia hataii	92.29	687	39	7	336	1010	1	685	0	963
bu_91849.1_c19034	gi|34555706	244861	Plerogyra sinuosa	91.25	686	47	10	336	1010	1	684	0	922
bu_91849.1_c19034	gi|34555708	46750	Euphyllia ancora	90.3	691	44	21	336	1010	1	684	0	883
bu_91849.1_c19034	gi|11118324	213639	Madrepora oculata	97.55	490	6	5	57	545	1	485	0	833
bu_91849.1_c19034	gi|11118326	213639	Madrepora oculata	95.43	481	21	1	620	1100	25	504	0	765
bu_91849.1_c19034	gi|11118326	213639	Madrepora oculata	98.45	194	3	0	1164	1357	505	698	4.00E-93	342
bu_91849.1_c19034	gi|77980189	46731	Pocillopora damicornis	99.76	411	1	0	1653	2063	1	411	0	754
bu_91849.1_c19034	gi|29565146	46731	Pocillopora damicornis	99.74	382	1	0	1679	2060	1	382	0	701
bu_91849.1_c19034	gi|29565146	46731	Pocillopora damicornis	99.74	382	1	0	1679	2060	1	382	0	701
bu_91849.1_c19034	gi|29565146	203993	Pocillopora verrucosa	98.97	388	1	1	1679	2063	1	388	0	691
bu_91849.1_c19034	gi|29565146	203993	Pocillopora verrucosa	98.97	388	1	1	1679	2063	1	388	0	691
bu_91849.1_c19034	gi|29565147	367768	Pocillopora eydouxi	96.73	397	1	3	1679	2063	1	397	0	651
bu_91849.1_c19034	gi|29565147	46732	Pocillopora meandrina	97.4	385	1	6	1679	2063	1	376	0	647
bu_91849.1_c19034	gi|29565147	367768	Pocillopora eydouxi	96.47	397	2	3	1679	2063	1	397	0	645
bu_91849.1_c19034	gi|29565147	46732	Pocillopora meandrina	97.14	385	2	6	1679	2063	1	376	0	641
bu_91849.1_c19034	gi|66391152	46731	Pocillopora damicornis	100	308	0	0	1756	2063	1	308	2.00E-161	569
bu_91849.1_c19034	gi|66391155	46731	Pocillopora damicornis	100	308	0	0	1756	2063	1	308	2.00E-161	569
bu_91849.1_c19034	gi|66391153	46731	Pocillopora damicornis	100	299	0	0	1765	2063	1	299	2.00E-156	553
bu_91849.1_c19034	gi|66391162	46731	Pocillopora damicornis	99.34	305	2	0	1756	2060	1	305	2.00E-156	553
bu_91849.1_c19034	gi|66391164	46731	Pocillopora damicornis	99.34	305	2	0	1756	2060	1	305	2.00E-156	553
bu_91849.1_c19034	gi|66391165	46731	Pocillopora damicornis	100	299	0	0	1765	2063	1	299	2.00E-156	553
bu_91849.1_c19034	gi|66391154	46731	Pocillopora damicornis	98.72	312	0	3	1756	2063	1	312	6.00E-156	551
bu_91849.1_c19034	gi|66391151	46731	Pocillopora damicornis	100	295	0	0	1769	2063	1	295	3.00E-154	545
bu_91849.1_c19034	gi|66391156	46731	Pocillopora damicornis	100	273	0	0	1791	2063	1	273	4.00E-142	505
bu_91849.1_c19034	gi|66391159	46731	Pocillopora damicornis	99.63	273	1	0	1791	2063	1	273	2.00E-140	499
bu_91849.1_c19034	gi|66391160	46731	Pocillopora damicornis	99.63	273	1	0	1791	2063	1	273	2.00E-140	499
bu_91849.1_c19034	gi|66391163	46731	Pocillopora damicornis	99.25	268	2	0	1793	2060	1	268	6.00E-136	484
bu_91849.1_c19034	gi|66391158	46731	Pocillopora damicornis	98.54	274	2	2	1791	2063	1	273	2.00E-135	483
bu_91849.1_c19034	gi|66391157	46731	Pocillopora damicornis	98.18	275	1	1	1793	2063	1	275	1.00E-133	477
bu_91849.1_c19034	gi|40018846	203993	Pocillopora verrucosa	97.15	281	3	5	1784	2063	1	277	1.00E-132	473
bu_91849.1_c19034	gi|66391161	46731	Pocillopora damicornis	96.38	276	6	4	1790	2063	1	274	6.00E-126	451
bu_91849.1_c19034	gi|22316068	203993	Pocillopora verrucosa	96.08	255	1	6	1809	2063	1	246	1.00E-112	407
bu_91849.1_c19034	gi|22278853	45264	Acropora millepora	85.75	393	29	26	270	649	155	533	1.00E-107	390
bu_91849.1_c19034	gi|22316070	203993	Pocillopora verrucosa	97.3	222	2	3	1835	2052	1	222	1.00E-102	374
bu_91849.1_c19034	gi|99963889	203993	Pocillopora verrucosa	94.81	231	6	6	1730	1958	57	283	5.00E-97	355
bu_91849.1_c19034	gi|11118328	213639	Madrepora oculata	98.45	194	3	0	1164	1357	1	194	4.00E-93	342
bu_91849.1_c19034	gi|22316069	203993	Pocillopora verrucosa	95.41	218	1	6	1835	2052	1	209	5.00E-92	339
bu_91849.1_c19034	gi|52547271	50429	Stylophora pistillata	97.86	187	1	3	1628	1813	5	189	2.00E-86	320

bu_91849.1_c19034	gi|33416022	174260	Lophelia pertusa	92.47	186	11	2	1628	1813	1	183	3.00E-69	263
bu_91849.1_c19034	gi|33416028	174260	Lophelia pertusa	92.47	186	11	2	1628	1813	7	189	3.00E-69	263
bu_91849.1_c19034	gi|33416043	174260	Lophelia pertusa	92.47	186	11	2	1628	1813	1	183	3.00E-69	263
bu_91849.1_c19034	gi|33416064	174260	Lophelia pertusa	92.47	186	11	2	1628	1813	4	186	3.00E-69	263
bu_91849.1_c19034	gi|33416025	174260	Lophelia pertusa	92.43	185	11	2	1629	1813	1	182	1.00E-68	261
bu_91849.1_c19034	gi|33416039	174260	Lophelia pertusa	92.43	185	11	2	1629	1813	1	182	1.00E-68	261
bu_91849.1_c19034	gi|33416027	174260	Lophelia pertusa	92.35	183	12	2	1631	1813	1	181	4.00E-68	259
bu_91849.1_c19034	gi|33416050	174260	Lophelia pertusa	91.98	187	11	3	1628	1813	3	186	4.00E-68	259
bu_91849.1_c19034	gi|33416019	174260	Lophelia pertusa	91.98	187	10	4	1628	1813	6	188	1.00E-67	257
bu_91849.1_c19034	gi|33416033	174260	Lophelia pertusa	92.35	183	11	2	1631	1813	1	180	1.00E-67	257
bu_91849.1_c19034	gi|33416034	174260	Lophelia pertusa	92.35	183	11	2	1631	1813	1	180	1.00E-67	257
bu_91849.1_c19034	gi|33416036	174260	Lophelia pertusa	92.35	183	11	2	1631	1813	1	180	1.00E-67	257
bu_91849.1_c19034	gi|33416092	174260	Lophelia pertusa	91.94	186	12	2	1628	1813	1	183	1.00E-67	257
bu_91849.1_c19034	gi|33416094	174260	Lophelia pertusa	92.35	183	11	2	1631	1813	1	180	1.00E-67	257
bu_91849.1_c19034	gi|33416042	174260	Lophelia pertusa	92.31	182	11	2	1632	1813	1	179	5.00E-67	255
bu_91849.1_c19034	gi|33416068	174260	Lophelia pertusa	91.58	190	8	7	1628	1813	3	188	5.00E-67	255
bu_91849.1_c19034	gi|33416049	174260	Lophelia pertusa	91.49	188	11	4	1628	1813	7	191	2.00E-66	254
bu_91849.1_c19034	gi|33416057	174260	Lophelia pertusa	91.49	188	11	3	1628	1813	7	191	2.00E-66	254
bu_91849.1_c19034	gi|33416023	174260	Lophelia pertusa	91.85	184	10	2	1631	1813	1	180	6.00E-66	252
bu_91849.1_c19034	gi|33416038	174260	Lophelia pertusa	91.8	183	12	2	1631	1813	1	180	6.00E-66	252
bu_91849.1_c19034	gi|33416044	174260	Lophelia pertusa	91.8	183	12	2	1631	1813	1	180	6.00E-66	252
bu_91849.1_c19034	gi|33416055	174260	Lophelia pertusa	91.44	187	11	4	1628	1813	1	183	6.00E-66	252
bu_91849.1_c19034	gi|33416060	174260	Lophelia pertusa	92.22	180	11	2	1634	1813	1	177	6.00E-66	252
bu_91849.1_c19034	gi|33416093	174260	Lophelia pertusa	92.22	180	11	2	1634	1813	1	177	6.00E-66	252
bu_91849.1_c19034	gi|52547272	293313	Tubastraea aurea	91.49	188	8	7	1628	1813	6	187	6.00E-66	252
bu_91849.1_c19034	gi|33416059	174260	Lophelia pertusa	91.1	191	7	8	1628	1813	6	191	2.00E-65	250
bu_91849.1_c19034	gi|52547267	293312	Stylocoeniella guentheri	95.03	161	5	2	1653	1813	1	158	2.00E-65	250
bu_91849.1_c19034	gi|52547268	293312	Stylocoeniella guentheri	95.03	161	5	2	1653	1813	1	158	2.00E-65	250
bu_91849.1_c19034	gi|52547269	293312	Stylocoeniella guentheri	95.03	161	5	2	1653	1813	1	158	2.00E-65	250
bu_91849.1_c19034	gi|33416020	174260	Lophelia pertusa	91.71	181	11	3	1634	1813	1	178	8.00E-65	248
bu_91849.1_c19034	gi|33416026	174260	Lophelia pertusa	91.71	181	11	2	1634	1813	1	178	8.00E-65	248
bu_91849.1_c19034	gi|33416029	174260	Lophelia pertusa	91.8	183	7	6	1634	1813	1	178	8.00E-65	248
bu_91849.1_c19034	gi|33416062	174260	Lophelia pertusa	91.71	181	11	3	1634	1813	1	178	8.00E-65	248
bu_91849.1_c19034	gi|33416063	174260	Lophelia pertusa	92.13	178	11	2	1636	1813	1	175	8.00E-65	248
bu_91849.1_c19034	gi|33416051	174260	Lophelia pertusa	91.67	180	11	2	1635	1813	1	177	3.00E-64	246
bu_91849.1_c19034	gi|33416032	174260	Lophelia pertusa	90.48	189	12	4	1628	1813	1	186	1.00E-63	244
bu_91849.1_c19034	gi|33416035	174260	Lophelia pertusa	90.86	186	11	3	1631	1813	1	183	1.00E-63	244
bu_91849.1_c19034	gi|33416018	174260	Lophelia pertusa	91.21	182	11	3	1634	1813	1	179	4.00E-63	243
bu_91849.1_c19034	gi|33416040	174260	Lophelia pertusa	90.37	187	14	3	1628	1813	6	189	4.00E-63	243
bu_91849.1_c19034	gi|33416056	174260	Lophelia pertusa	90.81	185	11	5	1633	1813	1	183	4.00E-63	243
bu_91849.1_c19034	gi|33416065	174260	Lophelia pertusa	91.11	180	13	1	1634	1813	1	177	1.00E-62	241
bu_91849.1_c19034	gi|33416021	174260	Lophelia pertusa	91.48	176	11	3	1639	1813	8	180	5.00E-62	239
bu_91849.1_c19034	gi|33416090	174260	Lophelia pertusa	91.91	173	11	2	1641	1813	1	170	5.00E-62	239
bu_91849.1_c19034	gi|29565147	50429	Stylophora pistillata	98.52	135	2	0	1679	1813	1	135	5.00E-62	239
bu_91849.1_c19034	gi|29565147	51070	Seriatopora hystrix	98.51	134	2	0	1679	1812	1	134	2.00E-61	237
bu_91849.1_c19034	gi|33416061	174260	Lophelia pertusa	91.01	178	11	4	1637	1813	8	181	6.00E-61	235
bu_91849.1_c19034	gi|20534526	123771	Madracis mirabilis	93.17	161	8	2	1653	1813	1	158	2.00E-60	233
bu_91849.1_c19034	gi|33416045	174260	Lophelia pertusa	89.89	188	9	8	1631	1813	1	183	2.00E-60	233
bu_91849.1_c19034	gi|33416052	174260	Lophelia pertusa	89.67	184	13	4	1632	1813	1	180	3.00E-59	230
bu_91849.1_c19034	gi|33416067	174260	Lophelia pertusa	89.73	185	11	8	1635	1813	1	183	3.00E-59	230
bu_91849.1_c19034	gi|56541598	50429	Stylophora pistillata	99.21	126	1	0	1688	1813	1	126	1.00E-58	228

<table>
<tr><td>bu_91849.1_c19034</td><td>gi|56541599</td><td>50429 Stylophora pistillata</td><td>99.21</td><td>126</td><td>1</td><td>0</td><td>1688</td><td>1813</td><td>1</td><td>126</td><td>1.00E-58</td><td>228</td></tr>
<tr><td>bu_91849.1_c19034</td><td>gi|56541600</td><td>50429 Stylophora pistillata</td><td>99.21</td><td>126</td><td>1</td><td>0</td><td>1688</td><td>1813</td><td>1</td><td>126</td><td>1.00E-58</td><td>228</td></tr>
<tr><td>bu_91849.1_c19034</td><td>gi|56541601</td><td>50429 Stylophora pistillata</td><td>99.21</td><td>126</td><td>1</td><td>0</td><td>1688</td><td>1813</td><td>1</td><td>126</td><td>1.00E-58</td><td>228</td></tr>
<tr><td>bu_91849.1_c19034</td><td>gi|56541602</td><td>50429 Stylophora pistillata</td><td>99.21</td><td>126</td><td>1</td><td>0</td><td>1688</td><td>1813</td><td>1</td><td>126</td><td>1.00E-58</td><td>228</td></tr>
<tr><td>bu_91849.1_c19034</td><td>gi|56541603</td><td>50429 Stylophora pistillata</td><td>99.21</td><td>126</td><td>1</td><td>0</td><td>1688</td><td>1813</td><td>1</td><td>126</td><td>1.00E-58</td><td>228</td></tr>
<tr><td>bu_91849.1_c19034</td><td>gi|56541604</td><td>50429 Stylophora pistillata</td><td>99.21</td><td>126</td><td>1</td><td>0</td><td>1688</td><td>1813</td><td>1</td><td>126</td><td>1.00E-58</td><td>228</td></tr>
<tr><td>bu_91849.1_c19034</td><td>gi|56541605</td><td>50429 Stylophora pistillata</td><td>99.21</td><td>126</td><td>1</td><td>0</td><td>1688</td><td>1813</td><td>1</td><td>126</td><td>1.00E-58</td><td>228</td></tr>
<tr><td>bu_91849.1_c19034</td><td>gi|56541606</td><td>50429 Stylophora pistillata</td><td>99.21</td><td>126</td><td>1</td><td>0</td><td>1688</td><td>1813</td><td>1</td><td>126</td><td>1.00E-58</td><td>228</td></tr>
<tr><td>bu_91849.1_c19034</td><td>gi|56541607</td><td>50429 Stylophora pistillata</td><td>99.21</td><td>126</td><td>1</td><td>0</td><td>1688</td><td>1813</td><td>1</td><td>126</td><td>1.00E-58</td><td>228</td></tr>
<tr><td>bu_91849.1_c19034</td><td>gi|56541608</td><td>50429 Stylophora pistillata</td><td>99.21</td><td>126</td><td>1</td><td>0</td><td>1688</td><td>1813</td><td>1</td><td>126</td><td>1.00E-58</td><td>228</td></tr>
<tr><td>bu_91849.1_c19034</td><td>gi|56541609</td><td>50429 Stylophora pistillata</td><td>99.21</td><td>126</td><td>1</td><td>0</td><td>1688</td><td>1813</td><td>1</td><td>126</td><td>1.00E-58</td><td>228</td></tr>
<tr><td>bu_91849.1_c19034</td><td>gi|56541610</td><td>50429 Stylophora pistillata</td><td>99.21</td><td>126</td><td>1</td><td>0</td><td>1688</td><td>1813</td><td>1</td><td>126</td><td>1.00E-58</td><td>228</td></tr>
<tr><td>bu_91849.1_c19034</td><td>gi|56541611</td><td>50429 Stylophora pistillata</td><td>99.21</td><td>126</td><td>1</td><td>0</td><td>1688</td><td>1813</td><td>1</td><td>126</td><td>1.00E-58</td><td>228</td></tr>
<tr><td>bu_91849.1_c19034</td><td>gi|56541612</td><td>50429 Stylophora pistillata</td><td>99.21</td><td>126</td><td>1</td><td>0</td><td>1688</td><td>1813</td><td>1</td><td>126</td><td>1.00E-58</td><td>228</td></tr>
<tr><td>bu_91849.1_c19034</td><td>gi|56541619</td><td>50429 Stylophora pistillata</td><td>99.21</td><td>126</td><td>1</td><td>0</td><td>1688</td><td>1813</td><td>1</td><td>126</td><td>1.00E-58</td><td>228</td></tr>
<tr><td>bu_91849.1_c19034</td><td>gi|56541620</td><td>50429 Stylophora pistillata</td><td>99.21</td><td>126</td><td>1</td><td>0</td><td>1688</td><td>1813</td><td>1</td><td>126</td><td>1.00E-58</td><td>228</td></tr>
<tr><td>bu_91849.1_c19034</td><td>gi|56541622</td><td>50429 Stylophora pistillata</td><td>99.21</td><td>126</td><td>1</td><td>0</td><td>1688</td><td>1813</td><td>1</td><td>126</td><td>1.00E-58</td><td>228</td></tr>
<tr><td>bu_91849.1_c19034</td><td>gi|56541623</td><td>50429 Stylophora pistillata</td><td>99.21</td><td>126</td><td>1</td><td>0</td><td>1688</td><td>1813</td><td>1</td><td>126</td><td>1.00E-58</td><td>228</td></tr>
<tr><td>bu_91849.1_c19034</td><td>gi|56541624</td><td>50429 Stylophora pistillata</td><td>99.21</td><td>126</td><td>1</td><td>0</td><td>1688</td><td>1813</td><td>1</td><td>126</td><td>1.00E-58</td><td>228</td></tr>
<tr><td>bu_91849.1_c19034</td><td>gi|56541625</td><td>50429 Stylophora pistillata</td><td>99.21</td><td>126</td><td>1</td><td>0</td><td>1688</td><td>1813</td><td>1</td><td>126</td><td>1.00E-58</td><td>228</td></tr>
<tr><td>bu_91849.1_c19034</td><td>gi|56541627</td><td>50429 Stylophora pistillata</td><td>99.21</td><td>126</td><td>1</td><td>0</td><td>1688</td><td>1813</td><td>1</td><td>126</td><td>1.00E-58</td><td>228</td></tr>
<tr><td>bu_91849.1_c19034</td><td>gi|56541628</td><td>50429 Stylophora pistillata</td><td>98.45</td><td>129</td><td>2</td><td>0</td><td>1688</td><td>1816</td><td>1</td><td>129</td><td>1.00E-58</td><td>228</td></tr>
<tr><td>bu_91849.1_c19034</td><td>gi|56541629</td><td>50429 Stylophora pistillata</td><td>99.21</td><td>126</td><td>1</td><td>0</td><td>1688</td><td>1813</td><td>1</td><td>126</td><td>1.00E-58</td><td>228</td></tr>
<tr><td>bu_91849.1_c19034</td><td>gi|56541630</td><td>50429 Stylophora pistillata</td><td>99.21</td><td>126</td><td>1</td><td>0</td><td>1688</td><td>1813</td><td>1</td><td>126</td><td>1.00E-58</td><td>228</td></tr>
<tr><td>bu_91849.1_c19034</td><td>gi|56541631</td><td>50429 Stylophora pistillata</td><td>99.21</td><td>126</td><td>1</td><td>0</td><td>1688</td><td>1813</td><td>1</td><td>126</td><td>1.00E-58</td><td>228</td></tr>
<tr><td>bu_91849.1_c19034</td><td>gi|56541632</td><td>50429 Stylophora pistillata</td><td>99.21</td><td>126</td><td>1</td><td>0</td><td>1688</td><td>1813</td><td>1</td><td>126</td><td>1.00E-58</td><td>228</td></tr>
<tr><td>bu_91849.1_c19034</td><td>gi|56541633</td><td>50429 Stylophora pistillata</td><td>99.21</td><td>126</td><td>1</td><td>0</td><td>1688</td><td>1813</td><td>1</td><td>126</td><td>1.00E-58</td><td>228</td></tr>
<tr><td>bu_91849.1_c19034</td><td>gi|56541634</td><td>50429 Stylophora pistillata</td><td>99.21</td><td>126</td><td>1</td><td>0</td><td>1688</td><td>1813</td><td>1</td><td>126</td><td>1.00E-58</td><td>228</td></tr>
<tr><td>bu_91849.1_c19034</td><td>gi|56541635</td><td>50429 Stylophora pistillata</td><td>99.21</td><td>126</td><td>1</td><td>0</td><td>1688</td><td>1813</td><td>1</td><td>126</td><td>1.00E-58</td><td>228</td></tr>
<tr><td>bu_91849.1_c19034</td><td>gi|56541636</td><td>50429 Stylophora pistillata</td><td>99.21</td><td>126</td><td>1</td><td>0</td><td>1688</td><td>1813</td><td>1</td><td>126</td><td>1.00E-58</td><td>228</td></tr>
<tr><td>bu_91849.1_c19034</td><td>gi|56541637</td><td>50429 Stylophora pistillata</td><td>99.21</td><td>126</td><td>1</td><td>0</td><td>1688</td><td>1813</td><td>1</td><td>126</td><td>1.00E-58</td><td>228</td></tr>
<tr><td>bu_91849.1_c19034</td><td>gi|56541638</td><td>50429 Stylophora pistillata</td><td>99.21</td><td>126</td><td>1</td><td>0</td><td>1688</td><td>1813</td><td>1</td><td>126</td><td>1.00E-58</td><td>228</td></tr>
<tr><td>bu_91849.1_c19034</td><td>gi|56541641</td><td>50429 Stylophora pistillata</td><td>99.21</td><td>126</td><td>1</td><td>0</td><td>1688</td><td>1813</td><td>1</td><td>126</td><td>1.00E-58</td><td>228</td></tr>
<tr><td>bu_91849.1_c19034</td><td>gi|56541642</td><td>50429 Stylophora pistillata</td><td>99.21</td><td>126</td><td>1</td><td>0</td><td>1688</td><td>1813</td><td>1</td><td>126</td><td>1.00E-58</td><td>228</td></tr>
<tr><td>bu_91849.1_c19034</td><td>gi|56541644</td><td>50429 Stylophora pistillata</td><td>99.21</td><td>126</td><td>1</td><td>0</td><td>1688</td><td>1813</td><td>1</td><td>126</td><td>1.00E-58</td><td>228</td></tr>
<tr><td>bu_91849.1_c19034</td><td>gi|82697098</td><td>50429 Stylophora pistillata</td><td>99.21</td><td>126</td><td>1</td><td>0</td><td>1688</td><td>1813</td><td>1</td><td>126</td><td>1.00E-58</td><td>228</td></tr>
<tr><td>bu_91849.1_c19034</td><td>gi|82697099</td><td>50429 Stylophora pistillata</td><td>99.21</td><td>126</td><td>1</td><td>0</td><td>1688</td><td>1813</td><td>1</td><td>126</td><td>1.00E-58</td><td>228</td></tr>
<tr><td>bu_91849.1_c19034</td><td>gi|82697100</td><td>50429 Stylophora pistillata</td><td>99.21</td><td>126</td><td>1</td><td>0</td><td>1688</td><td>1813</td><td>1</td><td>126</td><td>1.00E-58</td><td>228</td></tr>
<tr><td>bu_91849.1_c19034</td><td>gi|82697101</td><td>50429 Stylophora pistillata</td><td>99.21</td><td>126</td><td>1</td><td>0</td><td>1688</td><td>1813</td><td>1</td><td>126</td><td>1.00E-58</td><td>228</td></tr>
<tr><td>bu_91849.1_c19034</td><td>gi|82697102</td><td>50429 Stylophora pistillata</td><td>99.21</td><td>126</td><td>1</td><td>0</td><td>1688</td><td>1813</td><td>1</td><td>126</td><td>1.00E-58</td><td>228</td></tr>
<tr><td>bu_91849.1_c19034</td><td>gi|82697105</td><td>50429 Stylophora pistillata</td><td>99.21</td><td>126</td><td>1</td><td>0</td><td>1688</td><td>1813</td><td>1</td><td>126</td><td>1.00E-58</td><td>228</td></tr>
<tr><td>bu_91849.1_c19034</td><td>gi|82697107</td><td>50429 Stylophora pistillata</td><td>99.21</td><td>126</td><td>1</td><td>0</td><td>1688</td><td>1813</td><td>1</td><td>126</td><td>1.00E-58</td><td>228</td></tr>
<tr><td>bu_91849.1_c19034</td><td>gi|82697108</td><td>50429 Stylophora pistillata</td><td>99.21</td><td>126</td><td>1</td><td>0</td><td>1688</td><td>1813</td><td>1</td><td>126</td><td>1.00E-58</td><td>228</td></tr>
<tr><td>bu_91849.1_c19034</td><td>gi|82697109</td><td>50429 Stylophora pistillata</td><td>99.21</td><td>126</td><td>1</td><td>0</td><td>1688</td><td>1813</td><td>1</td><td>126</td><td>1.00E-58</td><td>228</td></tr>
<tr><td>bu_91849.1_c19034</td><td>gi|82697110</td><td>50429 Stylophora pistillata</td><td>99.21</td><td>126</td><td>1</td><td>0</td><td>1688</td><td>1813</td><td>1</td><td>126</td><td>1.00E-58</td><td>228</td></tr>
<tr><td>bu_91849.1_c19034</td><td>gi|82697111</td><td>50429 Stylophora pistillata</td><td>99.21</td><td>126</td><td>1</td><td>0</td><td>1688</td><td>1813</td><td>1</td><td>126</td><td>1.00E-58</td><td>228</td></tr>
<tr><td>bu_91849.1_c19034</td><td>gi|82697113</td><td>50429 Stylophora pistillata</td><td>99.21</td><td>126</td><td>1</td><td>0</td><td>1688</td><td>1813</td><td>1</td><td>126</td><td>1.00E-58</td><td>228</td></tr>
<tr><td>bu_91849.1_c19034</td><td>gi|82697118</td><td>50429 Stylophora pistillata</td><td>99.21</td><td>126</td><td>1</td><td>0</td><td>1688</td><td>1813</td><td>1</td><td>126</td><td>1.00E-58</td><td>228</td></tr>
<tr><td>bu_91849.1_c19034</td><td>gi|82697122</td><td>50429 Stylophora pistillata</td><td>99.21</td><td>126</td><td>1</td><td>0</td><td>1688</td><td>1813</td><td>1</td><td>126</td><td>1.00E-58</td><td>228</td></tr>
<tr><td>bu_91849.1_c19034</td><td>gi|82697123</td><td>50429 Stylophora pistillata</td><td>99.21</td><td>126</td><td>1</td><td>0</td><td>1688</td><td>1813</td><td>1</td><td>126</td><td>1.00E-58</td><td>228</td></tr>
<tr><td>bu_91849.1_c19034</td><td>gi|82697124</td><td>50429 Stylophora pistillata</td><td>99.21</td><td>126</td><td>1</td><td>0</td><td>1688</td><td>1813</td><td>1</td><td>126</td><td>1.00E-58</td><td>228</td></tr>
</table>

bu_91849.1_c19034	gi|82697125	50429	Stylophora pistillata	99.21	126	1	0	1688	1813	1	126	1.00E-58	228
bu_91849.1_c19034	gi|82697126	50429	Stylophora pistillata	99.21	126	1	0	1688	1813	1	126	1.00E-58	228
bu_91849.1_c19034	gi|82697127	50429	Stylophora pistillata	99.21	126	1	0	1688	1813	1	126	1.00E-58	228
bu_91849.1_c19034	gi|20534526	247026	Siderastrea stellata	92.55	161	9	2	1653	1813	1	158	1.00E-58	228
bu_91849.1_c19034	gi|52547250	214980	Montastrea curta	92.55	161	9	2	1653	1813	1	158	1.00E-58	228
bu_91849.1_c19034	gi|52547251	214980	Montastrea curta	92.55	161	9	2	1653	1813	1	158	1.00E-58	228
bu_91849.1_c19034	gi|52547245	154326	Hydnophora exesa	92.02	163	10	2	1653	1815	1	160	4.00E-58	226
bu_91849.1_c19034	gi|56541613	50429	Stylophora pistillata	98.43	127	1	1	1688	1813	1	127	5.00E-57	222
bu_91849.1_c19034	gi|56541614	50429	Stylophora pistillata	98.41	126	2	0	1688	1813	1	126	5.00E-57	222
bu_91849.1_c19034	gi|56541616	50429	Stylophora pistillata	98.41	126	2	0	1688	1813	1	126	5.00E-57	222
bu_91849.1_c19034	gi|56541617	50429	Stylophora pistillata	98.41	126	2	0	1688	1813	1	126	5.00E-57	222
bu_91849.1_c19034	gi|56541626	50429	Stylophora pistillata	98.41	126	2	0	1688	1813	1	126	5.00E-57	222
bu_91849.1_c19034	gi|56541640	50429	Stylophora pistillata	98.41	126	2	0	1688	1813	1	126	5.00E-57	222
bu_91849.1_c19034	gi|56541645	50429	Stylophora pistillata	98.41	126	2	0	1688	1813	1	126	5.00E-57	222
bu_91849.1_c19034	gi|82697103	50429	Stylophora pistillata	98.43	127	1	1	1688	1813	1	127	5.00E-57	222
bu_91849.1_c19034	gi|82697104	50429	Stylophora pistillata	98.43	127	1	1	1688	1813	1	127	5.00E-57	222
bu_91849.1_c19034	gi|82697106	50429	Stylophora pistillata	98.43	127	1	1	1688	1813	1	127	5.00E-57	222
bu_91849.1_c19034	gi|82697112	50429	Stylophora pistillata	98.43	127	1	1	1688	1813	1	127	5.00E-57	222
bu_91849.1_c19034	gi|20534525	187483	Plesiastrea versipora	91.98	162	8	2	1653	1813	1	158	5.00E-57	222
bu_91849.1_c19034	gi|20534525	51051	Acanthastrea echinata	91.93	161	10	2	1653	1813	1	158	5.00E-57	222
bu_91849.1_c19034	gi|20534525	214982	Mussa angulosa	91.93	161	10	2	1653	1813	1	158	5.00E-57	222
bu_91849.1_c19034	gi|20534525	419442	Physogyra lichtensteini	91.93	161	10	2	1653	1813	1	158	5.00E-57	222
bu_91849.1_c19034	gi|20534526	130191	Stephanocoenia michelinii	91.93	161	10	2	1653	1813	1	158	5.00E-57	222
bu_91849.1_c19034	gi|52547216	51051	Acanthastrea echinata	91.93	161	10	2	1653	1813	1	158	5.00E-57	222
bu_91849.1_c19034	gi|52547226	293182	Cyphastrea japonica	91.93	161	10	2	1653	1813	1	158	5.00E-57	222
bu_91849.1_c19034	gi|52547235	242764	Goniastrea aspera	91.93	161	10	2	1653	1813	1	158	5.00E-57	222
bu_91849.1_c19034	gi|52547236	242764	Goniastrea aspera	91.93	161	10	2	1653	1813	1	158	5.00E-57	222
bu_91849.1_c19034	gi|52547237	242764	Goniastrea aspera	91.93	161	10	2	1653	1813	1	158	5.00E-57	222
bu_91849.1_c19034	gi|52547242	293306	Goniastrea palauensis	91.93	161	10	2	1653	1813	1	158	5.00E-57	222
bu_91849.1_c19034	gi|52547265	51064	Pseudosiderastrea tayami	92.02	163	6	6	1653	1813	1	158	5.00E-57	222
bu_91849.1_c19034	gi|52547266	51064	Pseudosiderastrea tayami	92.02	163	6	6	1653	1813	1	158	5.00E-57	222
bu_91849.1_c19034	gi|82697116	50429	Stylophora pistillata	98.41	126	1	1	1688	1813	1	125	2.00E-56	220
bu_91849.1_c19034	gi|82697117	50429	Stylophora pistillata	99.18	122	1	0	1692	1813	3	124	2.00E-56	220
bu_91849.1_c19034	gi|52547246	154326	Hydnophora exesa	91.41	163	11	2	1653	1815	1	160	2.00E-56	220
bu_91849.1_c19034	gi|56541639	50429	Stylophora pistillata	97.62	126	3	0	1688	1813	1	126	2.00E-55	217
bu_91849.1_c19034	gi|20534525	214969	Diploastrea heliopora	91.3	161	11	2	1653	1813	1	158	2.00E-55	217
bu_91849.1_c19034	gi|20534526	465077	Gardineroseris planulata	91.36	162	9	3	1653	1813	1	158	2.00E-55	217
bu_91849.1_c19034	gi|20534526	541198	Goniopora columna	91.41	163	7	6	1653	1813	1	158	2.00E-55	217
bu_91849.1_c19034	gi|33416046	174260	Lophelia pertusa	91.3	161	11	2	1653	1813	1	158	2.00E-55	217
bu_91849.1_c19034	gi|33416047	174260	Lophelia pertusa	91.3	161	11	2	1653	1813	1	158	2.00E-55	217
bu_91849.1_c19034	gi|33416069	174260	Lophelia pertusa	91.3	161	11	2	1653	1813	1	158	2.00E-55	217
bu_91849.1_c19034	gi|33416070	174260	Lophelia pertusa	91.3	161	11	2	1653	1813	1	158	2.00E-55	217
bu_91849.1_c19034	gi|33416071	174260	Lophelia pertusa	91.3	161	11	2	1653	1813	1	158	2.00E-55	217
bu_91849.1_c19034	gi|33416072	174260	Lophelia pertusa	91.3	161	11	2	1653	1813	1	158	2.00E-55	217
bu_91849.1_c19034	gi|33416073	174260	Lophelia pertusa	91.3	161	11	2	1653	1813	1	158	2.00E-55	217
bu_91849.1_c19034	gi|33416077	174260	Lophelia pertusa	91.3	161	11	2	1653	1813	1	158	2.00E-55	217
bu_91849.1_c19034	gi|33416078	174260	Lophelia pertusa	91.3	161	11	2	1653	1813	1	158	2.00E-55	217
bu_91849.1_c19034	gi|33416079	174260	Lophelia pertusa	91.3	161	11	2	1653	1813	1	158	2.00E-55	217
bu_91849.1_c19034	gi|33416080	174260	Lophelia pertusa	91.3	161	11	2	1653	1813	1	158	2.00E-55	217
bu_91849.1_c19034	gi|33416081	174260	Lophelia pertusa	91.3	161	11	2	1653	1813	1	158	2.00E-55	217
bu_91849.1_c19034	gi|33416084	174260	Lophelia pertusa	91.3	161	11	2	1653	1813	1	158	2.00E-55	217

bu_91849.1_c19034	gi|33416085	174260	Lophelia pertusa	91.3	161	11	2	1653	1813	1	158	2.00E-55	217
bu_91849.1_c19034	gi|33416086	174260	Lophelia pertusa	91.3	161	11	2	1653	1813	1	158	2.00E-55	217
bu_91849.1_c19034	gi|33416088	174260	Lophelia pertusa	91.3	161	11	2	1653	1813	1	158	2.00E-55	217
bu_91849.1_c19034	gi|33416089	174260	Lophelia pertusa	91.3	161	11	2	1653	1813	1	158	2.00E-55	217
bu_91849.1_c19034	gi|33416091	174260	Lophelia pertusa	91.3	161	11	2	1653	1813	1	158	2.00E-55	217
bu_91849.1_c19034	gi|33416095	174260	Lophelia pertusa	91.3	161	11	2	1653	1813	1	158	2.00E-55	217
bu_91849.1_c19034	gi|52547215	51051	Acanthastrea echinata	91.3	161	11	2	1653	1813	1	158	2.00E-55	217
bu_91849.1_c19034	gi|52547225	293182	Cyphastrea japonica	91.3	161	11	2	1653	1813	1	158	2.00E-55	217
bu_91849.1_c19034	gi|52547227	293182	Cyphastrea japonica	91.3	161	11	2	1653	1813	1	158	2.00E-55	217
bu_91849.1_c19034	gi|52547231	126655	Favites abdita	91.36	162	9	4	1653	1813	1	158	2.00E-55	217
bu_91849.1_c19034	gi|52547232	126655	Favites abdita	91.36	162	9	4	1653	1813	1	158	2.00E-55	217
bu_91849.1_c19034	gi|52547238	293304	Goniastrea sp. CAC-2004	91.3	161	11	2	1653	1813	1	158	2.00E-55	217
bu_91849.1_c19034	gi|52547239	293304	Goniastrea sp. CAC-2004	91.3	161	11	2	1653	1813	1	158	2.00E-55	217
bu_91849.1_c19034	gi|52547258	213643	Psammocora contigua	91.3	161	11	2	1653	1813	1	158	2.00E-55	217
bu_91849.1_c19034	gi|52547259	213643	Psammocora contigua	91.3	161	11	2	1653	1813	1	158	2.00E-55	217
bu_91849.1_c19034	gi|52547262	51062	Porites lutea	91.41	163	7	6	1653	1813	1	158	2.00E-55	217
bu_91849.1_c19034	gi|52547263	51062	Porites lutea	91.41	163	7	6	1653	1813	1	158	2.00E-55	217
bu_91849.1_c19034	gi|52547264	51062	Porites lutea	91.41	163	7	6	1653	1813	1	158	2.00E-55	217
bu_91849.1_c19034	gi|20534526	126657	Pavona cactus	90.91	165	7	8	1653	1813	1	161	8.00E-55	215
bu_91849.1_c19034	gi|33416083	174260	Lophelia pertusa	91.25	160	11	2	1654	1813	1	157	8.00E-55	215
bu_91849.1_c19034	gi|22280191	45264	Acropora millepora	81.98	283	24	22	1423	1688	154	426	8.00E-55	215
bu_91849.1_c19034	gi|56541621	50429	Stylophora pistillata	97.58	124	3	0	1690	1813	3	126	3.00E-54	213
bu_91849.1_c19034	gi|56541643	50429	Stylophora pistillata	97.58	124	3	0	1690	1813	3	126	3.00E-54	213
bu_91849.1_c19034	gi|20534525	541204	Micromussa amakusensis	90.3	165	13	2	1653	1817	1	162	3.00E-54	213
bu_91849.1_c19034	gi|33416041	174260	Lophelia pertusa	91.19	159	11	2	1655	1813	1	156	3.00E-54	213
bu_91849.1_c19034	gi|33416087	174260	Lophelia pertusa	91.19	159	11	2	1655	1813	1	156	3.00E-54	213
bu_91849.1_c19034	gi|82697119	50429	Stylophora pistillata	98.35	121	1	1	1693	1813	6	125	1.00E-53	211
bu_91849.1_c19034	gi|82697120	50429	Stylophora pistillata	98.35	121	1	1	1693	1813	6	125	1.00E-53	211
bu_91849.1_c19034	gi|82697121	50429	Stylophora pistillata	98.35	121	1	1	1693	1813	6	125	1.00E-53	211
bu_91849.1_c19034	gi|20534525	258463	Solenastrea bournoni	90.68	161	12	2	1653	1813	1	158	1.00E-53	211
bu_91849.1_c19034	gi|20534526	371651	Coscinaraea columna	90.68	161	12	2	1653	1813	1	158	1.00E-53	211
bu_91849.1_c19034	gi|20534526	51068	Sandalolitha robusta	90.68	161	12	2	1653	1813	1	158	1.00E-53	211
bu_91849.1_c19034	gi|1809040|	51120	Scapophyllia cylindrica	90.68	161	12	2	1653	1813	1	158	1.00E-53	211
bu_91849.1_c19034	gi|52547229	293183	Cladocora sp. Kaohsiung CAC-2004	90.68	161	12	2	1653	1813	1	158	1.00E-53	211
bu_91849.1_c19034	gi|52547230	293183	Cladocora sp. Kaohsiung CAC-2004	90.68	161	12	2	1653	1813	1	158	1.00E-53	211
bu_91849.1_c19034	gi|52547260	213643	Psammocora contigua	90.74	162	10	4	1653	1813	1	158	1.00E-53	211
bu_91849.1_c19034	gi|56541618	50429	Stylophora pistillata	96.77	124	4	0	1690	1813	3	126	1.00E-52	207
bu_91849.1_c19034	gi|33416030	174260	Lophelia pertusa	91.03	156	11	2	1658	1813	1	153	1.00E-52	207
bu_91849.1_c19034	gi|33416082	174260	Lophelia pertusa	91.03	156	11	2	1658	1813	1	153	1.00E-52	207
bu_91849.1_c19034	gi|20534526	541197	Leptoseris yabei	90.12	162	11	4	1653	1813	1	158	5.00E-52	206
bu_91849.1_c19034	gi|52547228	293183	Cladocora sp. Kaohsiung CAC-2004	90.06	161	13	2	1653	1813	1	158	5.00E-52	206
bu_91849.1_c19034	gi|52547233	293184	Fungiacyathus sp. Ilan CAC-2004	89.82	167	8	8	1653	1813	1	164	5.00E-52	206
bu_91849.1_c19034	gi|52547234	293184	Fungiacyathus sp. Ilan CAC-2004	89.88	168	6	9	1653	1813	1	164	5.00E-52	206
bu_91849.1_c19034	gi|20534525	214969	Diploastrea heliopora	90.45	157	12	2	1657	1813	1	154	2.00E-51	204
bu_91849.1_c19034	gi|33416074	174260	Lophelia pertusa	90.91	154	11	2	1660	1813	1	151	2.00E-51	204
bu_91849.1_c19034	gi|52547243	293306	Goniastrea palauensis	91.39	151	10	2	1663	1813	1	148	2.00E-51	204
bu_91849.1_c19034	gi|82697115	50429	Stylophora pistillata	98.28	116	1	1	1698	1813	9	123	6.00E-51	202
bu_91849.1_c19034	gi|66391147	46731	Pocillopora damicornis	100	108	0	0	1956	2063	1	108	2.00E-50	200
bu_91849.1_c19034	gi|66391150	46731	Pocillopora damicornis	100	108	0	0	1956	2063	1	108	2.00E-50	200
bu_91849.1_c19034	gi|32347188	102205	Platygyra sinensis	91.78	146	9	2	1668	1813	1	143	2.00E-50	200
bu_91849.1_c19034	gi|32347185	154326	Hydnophora exesa	91.22	148	10	2	1668	1815	1	145	8.00E-50	198

bu_91849.1_c19034	gi|32347186	46760	Leptoria phrygia	90.73	151	10	3	1668	1817	1	148	8.00E-50	198
bu_91849.1_c19034	gi|32347182	498482	Favia maxima	91.67	144	9	2	1670	1813	1	141	3.00E-49	196
bu_91849.1_c19034	gi|32347183	570135	Favia rotumana	91.67	144	9	2	1670	1813	1	141	3.00E-49	196
bu_91849.1_c19034	gi|66391148	46731	Pocillopora damicornis	99.07	108	1	0	1956	2063	1	108	1.00E-48	195
bu_91849.1_c19034	gi|66391149	46731	Pocillopora damicornis	99.07	108	1	0	1956	2063	1	108	1.00E-48	195
bu_91849.1_c19034	gi|82697114	50429	Stylophora pistillata	99.08	109	0	1	1705	1813	17	124	1.00E-48	195
bu_91849.1_c19034	gi|14985023	46731	Pocillopora damicornis	99.07	108	1	0	1956	2063	1	108	1.00E-48	195
bu_91849.1_c19034	gi|52547244	293306	Goniastrea palauensis	91.1	146	10	2	1668	1813	1	143	1.00E-48	195
bu_91849.1_c19034	gi|16034761	126657	Pavona cactus	90.13	152	11	3	1667	1817	13	161	1.00E-48	195
bu_91849.1_c19034	gi|32347180	242776	Cyphastrea chalcidicum	91.1	146	10	2	1668	1813	1	143	1.00E-48	195
bu_91849.1_c19034	gi|32347181	102203	Favia favus	91.1	146	10	2	1668	1813	1	143	1.00E-48	195
bu_91849.1_c19034	gi|32347183	983568	Favia rotundata	91.61	143	9	2	1671	1813	1	140	1.00E-48	195
bu_91849.1_c19034	gi|32347184	983570	Favites stylifera	91.61	143	9	2	1671	1813	1	140	1.00E-48	195
bu_91849.1_c19034	gi|32347186	48500	Montastraea annularis	91.1	146	10	2	1668	1813	1	143	1.00E-48	195
bu_91849.1_c19034	gi|32347187	983583	Oulophyllia aff. bennettae DH-201	91.1	146	10	2	1668	1813	1	143	1.00E-48	195
bu_91849.1_c19034	gi|32347187	983578	Pectinia lactuca	91.1	146	10	2	1668	1813	1	143	1.00E-48	195
bu_91849.1_c19034	gi|32347189	196280	Trachyphyllia geoffroyi	90.6	149	11	2	1670	1818	1	146	1.00E-48	195
bu_91849.1_c19034	gi|32347183	983567	Favia rosaria	91.55	142	9	2	1672	1813	1	139	4.00E-48	193
bu_91849.1_c19034	gi|4028957|	50429	Stylophora pistillata	94.49	127	1	6	1688	1813	2	123	1.00E-47	191
bu_91849.1_c19034	gi|4028958|	50429	Stylophora pistillata	94.49	127	1	6	1688	1813	2	123	1.00E-47	191
bu_91849.1_c19034	gi|4028959|	50429	Stylophora pistillata	94.49	127	1	6	1688	1813	2	123	1.00E-47	191
bu_91849.1_c19034	gi|33416031	174260	Lophelia pertusa	88.75	160	13	4	1654	1813	1	155	1.00E-47	191
bu_91849.1_c19034	gi|33416076	174260	Lophelia pertusa	89.61	154	11	4	1661	1813	1	150	1.00E-47	191
bu_91849.1_c19034	gi|32347183	451375	Favia speciosa	90.97	144	10	2	1670	1813	1	141	1.00E-47	191
bu_91849.1_c19034	gi|32347184	242764	Goniastrea aspera	90.97	144	10	2	1670	1813	1	141	1.00E-47	191
bu_91849.1_c19034	gi|56541615	50429	Stylophora pistillata	93.65	126	8	0	1688	1813	1	126	5.00E-47	189
bu_91849.1_c19034	gi|32347180	187483	Plesiastrea versipora	90.97	144	8	2	1671	1813	1	140	5.00E-47	189
bu_91849.1_c19034	gi|32347181	498478	Favia danae	90.91	143	10	2	1671	1813	1	140	5.00E-47	189
bu_91849.1_c19034	gi|32347181	102203	Favia favus	90.91	143	10	2	1671	1813	1	140	5.00E-47	189
bu_91849.1_c19034	gi|32347182	498480	Favia lizardensis	90.91	143	10	2	1671	1813	1	140	5.00E-47	189
bu_91849.1_c19034	gi|32347182	498481	Favia matthaii	90.91	143	10	2	1671	1813	1	140	5.00E-47	189
bu_91849.1_c19034	gi|32347183	983586	Favia cf. maxima DH-2011	91.43	140	9	2	1674	1813	1	137	5.00E-47	189
bu_91849.1_c19034	gi|32347183	214973	Favia stelligera	90.41	146	11	2	1668	1813	1	143	5.00E-47	189
bu_91849.1_c19034	gi|32347186	983587	Montastraea cf. annuligera DH-201	90.91	143	10	2	1671	1813	1	140	5.00E-47	189
bu_91849.1_c19034	gi|32347183	242758	Favia pallida	90.85	142	10	2	1672	1813	1	139	2.00E-46	187
bu_91849.1_c19034	gi|32347187	983577	Mycedium robokaki	90.85	142	10	2	1672	1813	1	139	2.00E-46	187
bu_91849.1_c19034	gi|32347187	51058	Montastraea valenciennesi	90.34	145	9	4	1670	1813	2	142	6.00E-46	185
bu_91849.1_c19034	gi|32347181	242757	Echinopora gemmacea	90.21	143	11	2	1671	1813	1	140	2.00E-45	183
bu_91849.1_c19034	gi|32347185	465062	Goniastrea retiformis	89.73	146	12	2	1668	1813	1	143	2.00E-45	183
bu_91849.1_c19034	gi|11664255	375444	Platygyra sp. ZkYS1-27	91.73	133	8	2	1681	1813	6	135	8.00E-45	182
bu_91849.1_c19034	gi|32347183	451375	Favia speciosa	90.65	139	10	2	1675	1813	1	136	8.00E-45	182
bu_91849.1_c19034	gi|32347188	214987	Platygyra daedalea	91.73	133	8	2	1681	1813	2	131	8.00E-45	182
bu_91849.1_c19034	gi|32347188	242771	Platygyra lamellina	91.73	133	8	2	1681	1813	2	131	8.00E-45	182
bu_91849.1_c19034	gi|32347180	242754	Cyphastrea serailia	91.04	134	9	2	1680	1813	1	131	1.00E-43	178
bu_91849.1_c19034	gi|32347185	249004	Goniastrea favulus	89.51	143	12	2	1671	1813	1	140	1.00E-43	178
bu_91849.1_c19034	gi|33416024	174260	Lophelia pertusa	88.16	152	12	5	1665	1813	6	154	4.00E-43	176
bu_91849.1_c19034	gi|52547240	46745	Galaxea fascicularis	86.67	165	14	7	1653	1813	1	161	4.00E-43	176
bu_91849.1_c19034	gi|52547241	46745	Galaxea fascicularis	86.67	165	14	7	1653	1813	1	161	4.00E-43	176
bu_91849.1_c19034	gi|32347182	102203	Favia favus	90.98	133	9	2	1681	1813	1	130	4.00E-43	176
bu_91849.1_c19034	gi|32347182	498480	Favia lizardensis	90.98	133	9	2	1681	1813	2	131	4.00E-43	176
bu_91849.1_c19034	gi|32347182	983585	Favia cf. maritima DH-2011	90.98	133	9	2	1681	1813	4	133	4.00E-43	176

bu_91849.1_c19034	gi|52547270	51070	Seriatopora hystrix	98.98	98	0	1	1715	1812	41	137	1.00E-42	174
bu_91849.1_c19034	gi|32347184	498484	Favites flexuosa	90.3	134	10	2	1684	1817	1	131	5.00E-42	172
bu_91849.1_c19034	gi|33416037	174260	Lophelia pertusa	88.81	143	10	5	1673	1813	1	139	2.00E-41	171
bu_91849.1_c19034	gi|32347184	498483	Favites complanata	91.94	124	7	2	1690	1813	1	121	2.00E-41	171
bu_91849.1_c19034	gi|32347187	983591	Physophyllia ayleni	90.77	130	9	2	1684	1813	1	127	2.00E-41	171
bu_91849.1_c19034	gi|21311625	102205	Platygyra sinensis	96.12	103	4	0	1711	1813	13	115	7.00E-41	169
bu_91849.1_c19034	gi|21311626	102205	Platygyra sinensis	96.12	103	4	0	1711	1813	13	115	7.00E-41	169
bu_91849.1_c19034	gi|21311627	102205	Platygyra sinensis	96.12	103	4	0	1711	1813	13	115	7.00E-41	169
bu_91849.1_c19034	gi|21311629	102205	Platygyra sinensis	96.12	103	4	0	1711	1813	13	115	7.00E-41	169
bu_91849.1_c19034	gi|21311630	102205	Platygyra sinensis	96.12	103	4	0	1711	1813	13	115	7.00E-41	169
bu_91849.1_c19034	gi|21311631	102205	Platygyra sinensis	96.12	103	4	0	1711	1813	13	115	7.00E-41	169
bu_91849.1_c19034	gi|21311632	102205	Platygyra sinensis	96.12	103	4	0	1711	1813	13	115	7.00E-41	169
bu_91849.1_c19034	gi|21311633	102205	Platygyra sinensis	96.12	103	4	0	1711	1813	13	115	7.00E-41	169
bu_91849.1_c19034	gi|21311634	102205	Platygyra sinensis	96.12	103	4	0	1711	1813	13	115	7.00E-41	169
bu_91849.1_c19034	gi|21311635	102205	Platygyra sinensis	96.12	103	4	0	1711	1813	13	115	7.00E-41	169
bu_91849.1_c19034	gi|21311636	102205	Platygyra sinensis	96.12	103	4	0	1711	1813	13	115	7.00E-41	169
bu_91849.1_c19034	gi|21311637	102205	Platygyra sinensis	96.12	103	4	0	1711	1813	13	115	7.00E-41	169
bu_91849.1_c19034	gi|21311638	102205	Platygyra sinensis	96.12	103	4	0	1711	1813	13	115	7.00E-41	169
bu_91849.1_c19034	gi|21311639	102205	Platygyra sinensis	96.12	103	4	0	1711	1813	13	115	7.00E-41	169
bu_91849.1_c19034	gi|21311640	102205	Platygyra sinensis	96.12	103	4	0	1711	1813	13	115	7.00E-41	169
bu_91849.1_c19034	gi|21311641	102205	Platygyra sinensis	96.12	103	4	0	1711	1813	13	115	7.00E-41	169
bu_91849.1_c19034	gi|32347184	126655	Favites abdita	96.12	103	4	0	1711	1813	10	112	7.00E-41	169
bu_91849.1_c19034	gi|32347184	211865	Favites chinensis	96.12	103	4	0	1711	1813	20	122	7.00E-41	169
bu_91849.1_c19034	gi|32347184	498485	Favites paraflexuosa	96.12	103	4	0	1711	1813	8	110	7.00E-41	169
bu_91849.1_c19034	gi|32347184	983569	Favites russelli	96.12	103	4	0	1711	1813	7	109	7.00E-41	169
bu_91849.1_c19034	gi|32347185	983571	Hydnophora microconos	96.12	103	4	0	1711	1813	10	112	7.00E-41	169
bu_91849.1_c19034	gi|32347185	983572	Hydnophora pilosa	96.12	103	4	0	1711	1813	5	107	7.00E-41	169
bu_91849.1_c19034	gi|32347186	242769	Montastraea curta	96.12	103	4	0	1711	1813	12	114	7.00E-41	169
bu_91849.1_c19034	gi|32347188	983579	Platygyra acuta	96.12	103	4	0	1711	1813	10	112	7.00E-41	169
bu_91849.1_c19034	gi|32347188	983580	Platygyra contorta	96.12	103	4	0	1711	1813	10	112	7.00E-41	169
bu_91849.1_c19034	gi|32347188	194332	Platygyra pini	96.12	103	4	0	1711	1813	13	115	7.00E-41	169
bu_91849.1_c19034	gi|32347188	194332	Platygyra pini	96.12	103	4	0	1711	1813	9	111	7.00E-41	169
bu_91849.1_c19034	gi|32347188	983581	Platygyra ryukyuensis	96.12	103	4	0	1711	1813	6	108	7.00E-41	169
bu_91849.1_c19034	gi|32347188	983582	Platygyra cf. verweyi DH-2011	96.12	103	4	0	1711	1813	8	110	7.00E-41	169
bu_91849.1_c19034	gi|20534525	214970	Echinophyllia aspera	90	130	10	2	1684	1813	1	127	8.00E-40	165
bu_91849.1_c19034	gi|10709866	388437	Cladopsammia gracilis	97.89	95	2	0	1719	1813	7	101	8.00E-40	165
bu_91849.1_c19034	gi|10709866	388437	Cladopsammia gracilis	97.89	95	2	0	1719	1813	7	101	8.00E-40	165
bu_91849.1_c19034	gi|32347180	51051	Acanthastrea echinata	90	130	10	2	1684	1813	1	127	8.00E-40	165
bu_91849.1_c19034	gi|32347181	46694	Echinopora lamellosa	90	130	10	2	1684	1813	1	127	8.00E-40	165
bu_91849.1_c19034	gi|32347185	498487	Goniastrea australensis	90	130	10	2	1684	1813	1	127	8.00E-40	165
bu_91849.1_c19034	gi|32347186	983576	Montastraea salebrosa	90	130	10	2	1684	1813	1	127	8.00E-40	165
bu_91849.1_c19034	gi|11664255	375443	Platygyra sp. ZkYS1-b	95.15	103	5	0	1711	1813	7	109	3.00E-39	163
bu_91849.1_c19034	gi|10709865	388437	Cladopsammia gracilis	97.87	94	2	0	1720	1813	7	100	3.00E-39	163
bu_91849.1_c19034	gi|10709865	388437	Cladopsammia gracilis	97.87	94	2	0	1720	1813	7	100	3.00E-39	163
bu_91849.1_c19034	gi|10709865	388437	Cladopsammia gracilis	97.87	94	2	0	1720	1813	7	100	3.00E-39	163
bu_91849.1_c19034	gi|10709866	388437	Cladopsammia gracilis	97.87	94	2	0	1720	1813	7	100	3.00E-39	163
bu_91849.1_c19034	gi|10709866	388437	Cladopsammia gracilis	97.87	94	2	0	1720	1813	8	101	3.00E-39	163
bu_91849.1_c19034	gi|10709866	388437	Cladopsammia gracilis	97.87	94	2	0	1720	1813	7	100	3.00E-39	163
bu_91849.1_c19034	gi|32347180	541202	Barabattoia amicorum	90.48	126	9	2	1688	1813	1	123	3.00E-39	163
bu_91849.1_c19034	gi|32347181	102203	Favia favus	95.15	103	5	0	1711	1813	10	112	3.00E-39	163
bu_91849.1_c19034	gi|32347182	983584	Favia cf. laxa DH-2011	95.15	103	5	0	1711	1813	17	119	3.00E-39	163

bu_91849.1_c19034	gi|32347182	498481	Favia matthaii	95.15	103	5	0	1711	1813	17	119	3.00E-39	163
bu_91849.1_c19034	gi|32347182	498481	Favia matthaii	95.15	103	5	0	1711	1813	13	115	3.00E-39	163
bu_91849.1_c19034	gi|32347182	498481	Favia matthaii	95.15	103	5	0	1711	1813	11	113	3.00E-39	163
bu_91849.1_c19034	gi|32347183	242758	Favia pallida	95.15	103	5	0	1711	1813	13	115	3.00E-39	163
bu_91849.1_c19034	gi|32347183	865875	Favia truncatus	95.15	103	5	0	1711	1813	10	112	3.00E-39	163
bu_91849.1_c19034	gi|32347184	498486	Favites pentagona	95.15	103	5	0	1711	1813	15	117	3.00E-39	163
bu_91849.1_c19034	gi|32347185	293306	Goniastrea palauensis	95.15	103	5	0	1711	1813	20	122	3.00E-39	163
bu_91849.1_c19034	gi|32347186	51058	Montastraea valenciennesi	95.15	103	5	0	1711	1813	17	119	3.00E-39	163
bu_91849.1_c19034	gi|32347187	51060	Mycedium elephantotus	95.15	103	5	0	1711	1813	9	111	3.00E-39	163
bu_91849.1_c19034	gi|32347187	242768	Oulophyllia bennettae	95.15	103	5	0	1711	1813	20	122	3.00E-39	163
bu_91849.1_c19034	gi|32347187	242767	Oulophyllia crispa	95.15	103	5	0	1711	1813	10	112	3.00E-39	163
bu_91849.1_c19034	gi|32347187	46735	Pectinia alcicornis	95.15	103	5	0	1711	1813	12	114	3.00E-39	163
bu_91849.1_c19034	gi|2707902|	55976	Acropora valida	80.08	251	23	23	1454	1688	6	245	1.00E-38	161
bu_91849.1_c19034	gi|32347184	242760	Favites halicora	96.88	96	3	0	1718	1813	1	96	1.00E-38	161
bu_91849.1_c19034	gi|29087391	44304	Favia fragum	98.89	90	1	0	1724	1813	36	125	1.00E-38	161
bu_91849.1_c19034	gi|29087391	44304	Favia fragum	98.89	90	1	0	1724	1813	36	125	1.00E-38	161
bu_91849.1_c19034	gi|29087391	44304	Favia fragum	98.89	90	1	0	1724	1813	36	125	1.00E-38	161
bu_91849.1_c19034	gi|29087391	44304	Favia fragum	98.89	90	1	0	1724	1813	36	125	1.00E-38	161
bu_91849.1_c19034	gi|29087391	44304	Favia fragum	98.89	90	1	0	1724	1813	36	125	1.00E-38	161
bu_91849.1_c19034	gi|29087391	44304	Favia fragum	98.89	90	1	0	1724	1813	36	125	1.00E-38	161
bu_91849.1_c19034	gi|29087391	44304	Favia fragum	98.89	90	1	0	1724	1813	36	125	1.00E-38	161
bu_91849.1_c19034	gi|29087392	44304	Favia fragum	98.89	90	1	0	1724	1813	36	125	1.00E-38	161
bu_91849.1_c19034	gi|29087392	44304	Favia fragum	98.89	90	1	0	1724	1813	36	125	1.00E-38	161
bu_91849.1_c19034	gi|29087392	44304	Favia fragum	98.89	90	1	0	1724	1813	36	125	1.00E-38	161
bu_91849.1_c19034	gi|29087392	44304	Favia fragum	98.89	90	1	0	1724	1813	36	125	1.00E-38	161
bu_91849.1_c19034	gi|29087392	44304	Favia fragum	98.89	90	1	0	1724	1813	36	125	1.00E-38	161
bu_91849.1_c19034	gi|29087392	44304	Favia fragum	98.89	90	1	0	1724	1813	36	125	1.00E-38	161
bu_91849.1_c19034	gi|29087392	44304	Favia fragum	98.89	90	1	0	1724	1813	36	125	1.00E-38	161
bu_91849.1_c19034	gi|29087392	44304	Favia fragum	98.89	90	1	0	1724	1813	36	125	1.00E-38	161
bu_91849.1_c19034	gi|29087392	44304	Favia fragum	98.89	90	1	0	1724	1813	36	125	1.00E-38	161
bu_91849.1_c19034	gi|29087392	44304	Favia fragum	98.89	90	1	0	1724	1813	36	125	1.00E-38	161
bu_91849.1_c19034	gi|29087393	44304	Favia fragum	98.89	90	1	0	1724	1813	36	125	1.00E-38	161
bu_91849.1_c19034	gi|29087393	44304	Favia fragum	98.89	90	1	0	1724	1813	36	125	1.00E-38	161
bu_91849.1_c19034	gi|29087393	44304	Favia fragum	98.89	90	1	0	1724	1813	36	125	1.00E-38	161
bu_91849.1_c19034	gi|29087393	44304	Favia fragum	98.89	90	1	0	1724	1813	36	125	1.00E-38	161
bu_91849.1_c19034	gi|29087393	44304	Favia fragum	98.89	90	1	0	1724	1813	36	125	1.00E-38	161
bu_91849.1_c19034	gi|29087393	44304	Favia fragum	98.89	90	1	0	1724	1813	36	125	1.00E-38	161
bu_91849.1_c19034	gi|29087393	44304	Favia fragum	98.89	90	1	0	1724	1813	36	125	1.00E-38	161
bu_91849.1_c19034	gi|29087393	44304	Favia fragum	98.89	90	1	0	1724	1813	36	125	1.00E-38	161
bu_91849.1_c19034	gi|29087393	44304	Favia fragum	98.89	90	1	0	1724	1813	36	125	1.00E-38	161
bu_91849.1_c19034	gi|29087393	44304	Favia fragum	98.89	90	1	0	1724	1813	36	125	1.00E-38	161
bu_91849.1_c19034	gi|29087394	44304	Favia fragum	98.89	90	1	0	1724	1813	36	125	1.00E-38	161
bu_91849.1_c19034	gi|29087394	44304	Favia fragum	98.89	90	1	0	1724	1813	36	125	1.00E-38	161
bu_91849.1_c19034	gi|29087394	44304	Favia fragum	98.89	90	1	0	1724	1813	36	125	1.00E-38	161
bu_91849.1_c19034	gi|29087394	44304	Favia fragum	98.89	90	1	0	1724	1813	36	125	1.00E-38	161
bu_91849.1_c19034	gi|29087394	44304	Favia fragum	98.89	90	1	0	1724	1813	36	125	1.00E-38	161
bu_91849.1_c19034	gi|29087394	44304	Favia fragum	98.89	90	1	0	1724	1813	36	125	1.00E-38	161
bu_91849.1_c19034	gi|29087394	44304	Favia fragum	98.89	90	1	0	1724	1813	36	125	1.00E-38	161
bu_91849.1_c19034	gi|29087394	44304	Favia fragum	98.89	90	1	0	1724	1813	36	125	1.00E-38	161
bu_91849.1_c19034	gi|29087394	44304	Favia fragum	98.89	90	1	0	1724	1813	36	125	1.00E-38	161
bu_91849.1_c19034	gi|29087394	44304	Favia fragum	98.89	90	1	0	1724	1813	36	125	1.00E-38	161

bu_91849.1_c19034	gi|29087395	44304	Favia fragum	98.89	90	1	0	1724	1813	36	125	1.00E-38	161
bu_91849.1_c19034	gi|29087395	44304	Favia fragum	98.89	90	1	0	1724	1813	36	125	1.00E-38	161
bu_91849.1_c19034	gi|29087395	44304	Favia fragum	98.89	90	1	0	1724	1813	36	125	1.00E-38	161
bu_91849.1_c19034	gi|29087395	44304	Favia fragum	98.89	90	1	0	1724	1813	36	125	1.00E-38	161
bu_91849.1_c19034	gi|29087395	44304	Favia fragum	98.89	90	1	0	1724	1813	36	125	1.00E-38	161
bu_91849.1_c19034	gi|29087395	44304	Favia fragum	98.89	90	1	0	1724	1813	36	125	1.00E-38	161
bu_91849.1_c19034	gi|29087395	44304	Favia fragum	98.89	90	1	0	1724	1813	36	125	1.00E-38	161
bu_91849.1_c19034	gi|29087395	44304	Favia fragum	98.89	90	1	0	1724	1813	36	125	1.00E-38	161
bu_91849.1_c19034	gi|29087395	44304	Favia fragum	98.89	90	1	0	1724	1813	36	125	1.00E-38	161
bu_91849.1_c19034	gi|29087395	44304	Favia fragum	98.89	90	1	0	1724	1813	36	125	1.00E-38	161
bu_91849.1_c19034	gi|29087396	44304	Favia fragum	98.89	90	1	0	1724	1813	36	125	1.00E-38	161
bu_91849.1_c19034	gi|29087396	44304	Favia fragum	98.89	90	1	0	1724	1813	36	125	1.00E-38	161
bu_91849.1_c19034	gi|29087396	44304	Favia fragum	98.89	90	1	0	1724	1813	36	125	1.00E-38	161
bu_91849.1_c19034	gi|29087396	44304	Favia fragum	98.89	90	1	0	1724	1813	36	125	1.00E-38	161
bu_91849.1_c19034	gi|29087396	44304	Favia fragum	98.89	90	1	0	1724	1813	36	125	1.00E-38	161
bu_91849.1_c19034	gi|29087396	44304	Favia fragum	98.89	90	1	0	1724	1813	36	125	1.00E-38	161
bu_91849.1_c19034	gi|29087396	44304	Favia fragum	98.89	90	1	0	1724	1813	36	125	1.00E-38	161
bu_91849.1_c19034	gi|29087396	44304	Favia fragum	98.89	90	1	0	1724	1813	36	125	1.00E-38	161
bu_91849.1_c19034	gi|29087396	44304	Favia fragum	98.89	90	1	0	1724	1813	36	125	1.00E-38	161
bu_91849.1_c19034	gi|29087396	44304	Favia fragum	98.89	90	1	0	1724	1813	36	125	1.00E-38	161
bu_91849.1_c19034	gi|29087397	44304	Favia fragum	98.89	90	1	0	1724	1813	36	125	1.00E-38	161
bu_91849.1_c19034	gi|29087397	44304	Favia fragum	98.89	90	1	0	1724	1813	36	125	1.00E-38	161
bu_91849.1_c19034	gi|29087397	44304	Favia fragum	98.89	90	1	0	1724	1813	36	125	1.00E-38	161
bu_91849.1_c19034	gi|29087397	44304	Favia fragum	98.89	90	1	0	1724	1813	36	125	1.00E-38	161
bu_91849.1_c19034	gi|29087397	44304	Favia fragum	98.89	90	1	0	1724	1813	36	125	1.00E-38	161
bu_91849.1_c19034	gi|29087397	44304	Favia fragum	98.89	90	1	0	1724	1813	36	125	1.00E-38	161
bu_91849.1_c19034	gi|29087397	44304	Favia fragum	98.89	90	1	0	1724	1813	36	125	1.00E-38	161
bu_91849.1_c19034	gi|29087397	44304	Favia fragum	98.89	90	1	0	1724	1813	36	125	1.00E-38	161
bu_91849.1_c19034	gi|29087397	44304	Favia fragum	98.89	90	1	0	1724	1813	36	125	1.00E-38	161
bu_91849.1_c19034	gi|29087397	44304	Favia fragum	98.89	90	1	0	1724	1813	36	125	1.00E-38	161
bu_91849.1_c19034	gi|29087398	44304	Favia fragum	98.89	90	1	0	1724	1813	36	125	1.00E-38	161
bu_91849.1_c19034	gi|29087398	44304	Favia fragum	98.89	90	1	0	1724	1813	36	125	1.00E-38	161
bu_91849.1_c19034	gi|29087398	44304	Favia fragum	98.89	90	1	0	1724	1813	36	125	1.00E-38	161
bu_91849.1_c19034	gi|29087398	44304	Favia fragum	98.89	90	1	0	1724	1813	36	125	1.00E-38	161
bu_91849.1_c19034	gi|29087398	44304	Favia fragum	98.89	90	1	0	1724	1813	36	125	1.00E-38	161
bu_91849.1_c19034	gi|29087398	44304	Favia fragum	98.89	90	1	0	1724	1813	36	125	1.00E-38	161
bu_91849.1_c19034	gi|29087398	44304	Favia fragum	98.89	90	1	0	1724	1813	36	125	1.00E-38	161
bu_91849.1_c19034	gi|29087398	44304	Favia fragum	98.89	90	1	0	1724	1813	36	125	1.00E-38	161
bu_91849.1_c19034	gi|29087398	44304	Favia fragum	98.89	90	1	0	1724	1813	36	125	1.00E-38	161
bu_91849.1_c19034	gi|29087398	44304	Favia fragum	98.89	90	1	0	1724	1813	36	125	1.00E-38	161
bu_91849.1_c19034	gi|29087399	44304	Favia fragum	98.89	90	1	0	1724	1813	36	125	1.00E-38	161
bu_91849.1_c19034	gi|29087399	44304	Favia fragum	98.89	90	1	0	1724	1813	36	125	1.00E-38	161
bu_91849.1_c19034	gi|29087399	44304	Favia fragum	98.89	90	1	0	1724	1813	36	125	1.00E-38	161
bu_91849.1_c19034	gi|29087399	44304	Favia fragum	98.89	90	1	0	1724	1813	36	125	1.00E-38	161
bu_91849.1_c19034	gi|29087399	44304	Favia fragum	98.89	90	1	0	1724	1813	36	125	1.00E-38	161
bu_91849.1_c19034	gi|29087399	44304	Favia fragum	98.89	90	1	0	1724	1813	36	125	1.00E-38	161
bu_91849.1_c19034	gi|29087399	44304	Favia fragum	98.89	90	1	0	1724	1813	36	125	1.00E-38	161
bu_91849.1_c19034	gi|29087399	44304	Favia fragum	98.89	90	1	0	1724	1813	36	125	1.00E-38	161
bu_91849.1_c19034	gi|29087399	44304	Favia fragum	98.89	90	1	0	1724	1813	36	125	1.00E-38	161
bu_91849.1_c19034	gi|29087399	44304	Favia fragum	98.89	90	1	0	1724	1813	36	125	1.00E-38	161
bu_91849.1_c19034	gi|29087400	44304	Favia fragum	98.89	90	1	0	1724	1813	36	125	1.00E-38	161

bu_91849.1_c19034	gi|29087400	44304	Favia fragum	98.89	90	1	0	1724	1813	36	125	1.00E-38	161
bu_91849.1_c19034	gi|29087400	44304	Favia fragum	98.89	90	1	0	1724	1813	36	125	1.00E-38	161
bu_91849.1_c19034	gi|20534525	214986	Oculina diffusa	89.23	130	11	2	1684	1813	7	133	4.00E-38	159
bu_91849.1_c19034	gi|32347186	63558	Montastraea cavernosa	89.76	127	10	2	1687	1813	1	124	4.00E-38	159
bu_91849.1_c19034	gi|2707901|	45264	Acropora millepora	79.76	252	23	23	1454	1688	6	246	1.00E-37	158
bu_91849.1_c19034	gi|21311628	102205	Platygyra sinensis	94.17	103	6	0	1711	1813	13	115	1.00E-37	158
bu_91849.1_c19034	gi|33416075	174260	Lophelia pertusa	88.72	133	10	4	1683	1813	4	133	1.00E-37	158
bu_91849.1_c19034	gi|16034761	126657	Pavona cactus	95.92	98	3	1	1720	1817	20	116	1.00E-37	158
bu_91849.1_c19034	gi|16034761	126657	Pavona cactus	95.92	98	3	1	1720	1817	24	120	1.00E-37	158
bu_91849.1_c19034	gi|16034761	126657	Pavona cactus	95.92	98	3	1	1720	1817	26	122	1.00E-37	158
bu_91849.1_c19034	gi|16034762	126657	Pavona cactus	95.92	98	3	1	1720	1817	25	121	1.00E-37	158
bu_91849.1_c19034	gi|16034762	126657	Pavona cactus	95.92	98	3	1	1720	1817	24	120	1.00E-37	158
bu_91849.1_c19034	gi|32347180	242776	Cyphastrea chalcidicum	96.81	94	3	0	1720	1813	18	111	1.00E-37	158
bu_91849.1_c19034	gi|32347180	242754	Cyphastrea serailia	96.81	94	3	0	1720	1813	22	115	1.00E-37	158
bu_91849.1_c19034	gi|32347181	214969	Diploastrea heliopora	96.81	94	3	0	1720	1813	19	112	1.00E-37	158
bu_91849.1_c19034	gi|32347181	983564	Echinopora horrida	96.81	94	3	0	1720	1813	19	112	1.00E-37	158
bu_91849.1_c19034	gi|32347181	983565	Echinopora mammiformis	96.81	94	3	0	1720	1813	23	116	1.00E-37	158
bu_91849.1_c19034	gi|32347181	242756	Echinopora pacificus	96.81	94	3	0	1720	1813	21	114	1.00E-37	158
bu_91849.1_c19034	gi|32347185	498487	Goniastrea australensis	96.81	94	3	0	1720	1813	11	104	1.00E-37	158
bu_91849.1_c19034	gi|32347185	498488	Goniastrea edwardsi	96.81	94	3	0	1720	1813	19	112	1.00E-37	158
bu_91849.1_c19034	gi|32347185	242763	Goniastrea pectinata	96.81	94	3	0	1720	1813	26	119	1.00E-37	158
bu_91849.1_c19034	gi|32347186	46738	Merulina scabricula	96.81	94	3	0	1720	1813	19	112	1.00E-37	158
bu_91849.1_c19034	gi|32347187	983589	Moseleya latistellata	96.81	94	3	0	1720	1813	19	112	1.00E-37	158
bu_91849.1_c19034	gi|32347189	51120	Scapophyllia cylindrica	96.81	94	3	0	1720	1813	18	111	1.00E-37	158
bu_91849.1_c19034	gi|16034761	126657	Pavona cactus	95	100	4	1	1719	1817	8	107	5.00E-37	156
bu_91849.1_c19034	gi|16034762	227230	Pavona decussata	96.81	94	2	1	1720	1813	26	118	5.00E-37	156
bu_91849.1_c19034	gi|2707900|	55975	Acropora longicyathus	79.76	247	22	24	1454	1683	6	241	2.00E-36	154
bu_91849.1_c19034	gi|4028951|	75301	Goniopora tenuidens	94.9	98	3	2	1720	1817	34	129	7.00E-36	152
bu_91849.1_c19034	gi|4028952|	75301	Goniopora tenuidens	94.9	98	3	2	1720	1817	25	120	7.00E-36	152
bu_91849.1_c19034	gi|32347180	983563	Caulastraea tumida	93.2	103	7	0	1711	1813	10	112	7.00E-36	152
bu_91849.1_c19034	gi|21311642	194332	Platygyra pini	98.81	84	1	0	1730	1813	2	85	2.00E-35	150
bu_91849.1_lrc19340	gi|58865331	46731	Pocillopora damicornis	99.25	938	4	3	11	947	959	1894	0	1690
bu_91849.1_lrc19340	gi|6465981|	50429	Stylophora pistillata	91.29	941	73	9	11	947	2791	3726	0	1275
bu_91849.1_c19814	gi|15443469	46731	Pocillopora damicornis	98.5	1399	21	0	293	1691	1	1399	0	2486
bu_91849.1_c22592	gi|22449800	46731	Pocillopora damicornis	96.08	740	20	6	32	767	11	745	0	1199
bu_91849.1_c23199	gi|22449800	46731	Pocillopora damicornis	94.12	612	19	15	74	677	97	699	0	920
bu_91849.1_c26383	gi|17029716	46732	Pocillopora meandrina	82.86	140	24	0	49	188	283	422	2.00E-28	126
bu_91849.1_c26383	gi|17029717	46732	Pocillopora meandrina	82.86	140	24	0	49	188	283	422	2.00E-28	126
bu_91849.1_c26383	gi|17029717	46732	Pocillopora meandrina	82.86	140	24	0	49	188	283	422	2.00E-28	126
bu_91849.1_c26383	gi|17029712	46731	Pocillopora damicornis	82.14	140	25	0	49	188	283	422	1.00E-26	121
bu_91849.1_c26383	gi|17029712	46731	Pocillopora damicornis	82.14	140	25	0	49	188	283	422	1.00E-26	121
bu_91849.1_c26383	gi|17029713	46731	Pocillopora damicornis	82.14	140	25	0	49	188	283	422	1.00E-26	121
bu_91849.1_c26383	gi|17029713	46731	Pocillopora damicornis	82.14	140	25	0	49	188	283	422	1.00E-26	121
bu_91849.1_c26383	gi|17029713	46731	Pocillopora damicornis	82.14	140	25	0	49	188	283	422	1.00E-26	121
bu_91849.1_c26383	gi|17029713	46731	Pocillopora damicornis	82.14	140	25	0	49	188	283	422	1.00E-26	121
bu_91849.1_c26383	gi|17029713	46731	Pocillopora damicornis	82.14	140	25	0	49	188	283	422	1.00E-26	121
bu_91849.1_c26383	gi|17029714	46731	Pocillopora damicornis	82.14	140	25	0	49	188	283	422	1.00E-26	121
bu_91849.1_c26383	gi|17029714	46731	Pocillopora damicornis	82.14	140	25	0	49	188	329	468	1.00E-26	121
bu_91849.1_c26383	gi|17029714	46731	Pocillopora damicornis	82.14	140	25	0	49	188	283	422	1.00E-26	121
bu_91849.1_c26383	gi|17029714	46731	Pocillopora damicornis	82.14	140	25	0	49	188	283	422	1.00E-26	121
bu_91849.1_c26383	gi|17029714	46731	Pocillopora damicornis	82.14	140	25	0	49	188	329	468	1.00E-26	121

bu_91849.1_c26383	gi|17029714	46731	Pocillopora damicornis	82.14	140	25	0	49	188	283	422	1.00E-26	121
bu_91849.1_c26383	gi|17029715	46731	Pocillopora damicornis	82.14	140	25	0	49	188	329	468	1.00E-26	121
bu_91849.1_c26383	gi|17029715	46731	Pocillopora damicornis	82.14	140	25	0	49	188	329	468	1.00E-26	121
bu_91849.1_c26383	gi|17029716	46731	Pocillopora damicornis	82.14	140	25	0	49	188	329	468	1.00E-26	121
bu_91849.1_c26383	gi|17029716	46731	Pocillopora damicornis	82.14	140	25	0	49	188	283	422	1.00E-26	121
bu_91849.1_c26383	gi|17029716	46731	Pocillopora damicornis	82.14	140	25	0	49	188	329	468	1.00E-26	121
bu_91849.1_c26383	gi|17029716	46732	Pocillopora meandrina	82.14	140	25	0	49	188	283	422	1.00E-26	121
bu_91849.1_c26383	gi|17029717	46732	Pocillopora meandrina	82.14	140	25	0	49	188	283	422	1.00E-26	121
bu_91849.1_c26383	gi|17029717	512010	Pocillopora ligulata	82.14	140	25	0	49	188	283	422	1.00E-26	121
bu_91849.1_c26383	gi|17029718	512010	Pocillopora ligulata	82.14	140	25	0	49	188	283	422	1.00E-26	121
bu_91849.1_c26383	gi|17029718	512010	Pocillopora ligulata	82.14	140	25	0	49	188	283	422	1.00E-26	121
bu_91849.1_c26383	gi|17029718	512010	Pocillopora ligulata	82.14	140	25	0	49	188	283	422	1.00E-26	121
bu_91849.1_c26383	gi|17029718	512010	Pocillopora ligulata	82.14	140	25	0	49	188	283	422	1.00E-26	121
bu_91849.1_c26383	gi|17029718	512010	Pocillopora ligulata	82.14	140	25	0	49	188	283	422	1.00E-26	121
bu_91849.1_c26383	gi|17029718	367768	Pocillopora eydouxi	82.14	140	25	0	49	188	269	408	1.00E-26	121
bu_91849.1_c26383	gi|17029715	46731	Pocillopora damicornis	81.75	137	25	0	49	185	283	419	5.00E-25	115
bu_91849.1_c26383	gi|17029717	512010	Pocillopora ligulata	81.43	140	26	0	49	188	283	422	5.00E-25	115
bu_91849.1_c26383	gi|17029718	512010	Pocillopora ligulata	81.43	140	26	0	49	188	283	422	5.00E-25	115
bu_91849.1_c26383	gi|17029716	46732	Pocillopora meandrina	80.85	141	25	2	49	188	284	423	2.00E-23	110
bu_91849.1_c26383	gi|17029716	46732	Pocillopora meandrina	80.85	141	25	2	49	188	284	423	2.00E-23	110
bu_91849.1_c26383	gi|17029717	46732	Pocillopora meandrina	80.85	141	25	2	49	188	284	423	2.00E-23	110
bu_91849.1_c26383	gi|17029717	46732	Pocillopora meandrina	80.85	141	25	2	49	188	284	423	2.00E-23	110
bu_91849.1_c26383	gi|17029718	367768	Pocillopora eydouxi	80.85	141	25	2	49	188	284	423	2.00E-23	110
bu_91849.1_c26383	gi|17029718	367768	Pocillopora eydouxi	80.85	141	25	2	49	188	284	423	2.00E-23	110
bu_91849.1_c26383	gi|17029719	367768	Pocillopora eydouxi	80.85	141	25	2	49	188	284	423	2.00E-23	110
bu_91849.1_c26383	gi|17029718	367768	Pocillopora eydouxi	80.71	140	26	1	49	188	265	403	9.00E-23	108
bu_91849.1_c26383	gi|17029719	367768	Pocillopora eydouxi	80.71	140	26	1	49	188	265	403	9.00E-23	108
bu_91849.1_c26383	gi|17029719	512011	Pocillopora molokensis	80.71	140	26	1	49	188	265	403	9.00E-23	108
bu_91849.1_c26383	gi|17029719	512011	Pocillopora molokensis	80.71	140	26	1	49	188	265	403	9.00E-23	108
bu_91849.1_c26383	gi|17029719	512011	Pocillopora molokensis	80.71	140	26	1	49	188	265	403	9.00E-23	108
bu_91849.1_c26383	gi|17029719	512011	Pocillopora molokensis	80.71	140	26	1	49	188	265	403	9.00E-23	108
bu_91849.1_c26383	gi|17029719	512011	Pocillopora molokensis	80.71	140	26	1	49	188	265	403	9.00E-23	108
bu_91849.1_c26383	gi|17029719	512011	Pocillopora molokensis	80.71	140	26	1	49	188	265	403	9.00E-23	108
bu_91849.1_c26383	gi|17029719	512011	Pocillopora molokensis	80.71	140	26	1	49	188	265	403	9.00E-23	108
bu_91849.1_c26383	gi|17029719	512011	Pocillopora molokensis	80.71	140	26	1	49	188	265	403	9.00E-23	108
bu_91849.1_c26383	gi|17029720	512011	Pocillopora molokensis	80.71	140	26	1	49	188	265	403	9.00E-23	108
bu_91849.1_c26383	gi|17029720	512011	Pocillopora molokensis	80.71	140	26	1	49	188	265	403	9.00E-23	108
bu_91849.1_c26383	gi|17029712	46731	Pocillopora damicornis	80	140	27	1	49	188	265	403	4.00E-21	102
bu_91849.1_c26383	gi|17029713	46731	Pocillopora damicornis	80	140	27	1	49	188	265	403	4.00E-21	102
bu_91849.1_c26383	gi|17029713	46731	Pocillopora damicornis	80	140	27	1	49	188	283	421	4.00E-21	102
bu_91849.1_c26383	gi|17029713	46731	Pocillopora damicornis	80	140	27	1	49	188	265	403	4.00E-21	102
bu_91849.1_c26383	gi|17029713	46731	Pocillopora damicornis	80	140	27	1	49	188	283	421	4.00E-21	102
bu_91849.1_c26383	gi|17029713	46731	Pocillopora damicornis	80	140	27	1	49	188	265	403	4.00E-21	102
bu_91849.1_c26383	gi|17029714	46731	Pocillopora damicornis	80	140	27	1	49	188	265	403	4.00E-21	102
bu_91849.1_c26383	gi|17029714	46731	Pocillopora damicornis	80	140	27	1	49	188	265	403	4.00E-21	102
bu_91849.1_c26383	gi|17029714	46731	Pocillopora damicornis	80	140	27	1	49	188	265	403	4.00E-21	102
bu_91849.1_c26383	gi|17029714	46731	Pocillopora damicornis	80	140	27	1	49	188	265	403	4.00E-21	102
bu_91849.1_c26383	gi|17029715	46731	Pocillopora damicornis	80	140	27	1	49	188	265	403	4.00E-21	102
bu_91849.1_c26383	gi|17029715	46731	Pocillopora damicornis	80	140	27	1	49	188	265	403	4.00E-21	102
bu_91849.1_c26383	gi|17029715	46731	Pocillopora damicornis	80	140	27	1	49	188	265	403	4.00E-21	102
bu_91849.1_c26383	gi|17029715	46731	Pocillopora damicornis	80	140	27	1	49	188	265	403	4.00E-21	102

bu_91849.1_c26383	gi|17029715	46731	Pocillopora damicornis	80	140	27	1	49	188	265	403	4.00E-21	102
bu_91849.1_c26383	gi|17029716	46731	Pocillopora damicornis	80	140	27	1	49	188	265	403	4.00E-21	102
bu_91849.1_c26383	gi|17029716	46731	Pocillopora damicornis	80	140	27	1	49	188	265	403	4.00E-21	102
bu_91849.1_c26383	gi|17029716	46732	Pocillopora meandrina	79.29	140	29	0	49	188	283	422	5.00E-20	99
bu_91849.1_c26383	gi|17029717	46732	Pocillopora meandrina	79.29	140	29	0	49	188	283	422	5.00E-20	99
bu_91849.1_c26383	gi|17029717	46732	Pocillopora meandrina	79.29	140	29	0	49	188	283	422	5.00E-20	99
bu_91849.1_c26383	gi|17029717	46732	Pocillopora meandrina	79.29	140	29	0	49	188	283	422	5.00E-20	99
bu_91849.1_lrc26991	gi|17029713	46731	Pocillopora damicornis	84.85	165	20	5	491	652	280	442	4.00E-39	161
bu_91849.1_lrc26991	gi|17029713	46731	Pocillopora damicornis	84.85	165	20	5	491	652	280	442	4.00E-39	161
bu_91849.1_lrc26991	gi|17029714	46731	Pocillopora damicornis	84.85	165	20	5	491	652	280	442	4.00E-39	161
bu_91849.1_lrc26991	gi|17029714	46731	Pocillopora damicornis	84.85	165	20	5	491	652	280	442	4.00E-39	161
bu_91849.1_lrc26991	gi|17029716	46732	Pocillopora meandrina	84.76	164	22	3	491	652	280	442	4.00E-39	161
bu_91849.1_lrc26991	gi|17029717	46732	Pocillopora meandrina	84.76	164	22	3	491	652	280	442	4.00E-39	161
bu_91849.1_lrc26991	gi|17029717	46732	Pocillopora meandrina	84.76	164	22	3	491	652	280	442	4.00E-39	161
bu_91849.1_lrc26991	gi|17029712	46731	Pocillopora damicornis	84.15	164	23	3	491	652	280	442	2.00E-37	156
bu_91849.1_lrc26991	gi|17029712	46731	Pocillopora damicornis	84.15	164	23	3	491	652	280	442	2.00E-37	156
bu_91849.1_lrc26991	gi|17029713	46731	Pocillopora damicornis	84.15	164	23	3	491	652	280	442	2.00E-37	156
bu_91849.1_lrc26991	gi|17029713	46731	Pocillopora damicornis	84.15	164	23	3	491	652	280	442	2.00E-37	156
bu_91849.1_lrc26991	gi|17029713	46731	Pocillopora damicornis	84.15	164	23	3	491	652	280	442	2.00E-37	156
bu_91849.1_lrc26991	gi|17029714	46731	Pocillopora damicornis	84.15	164	23	3	491	652	280	442	2.00E-37	156
bu_91849.1_lrc26991	gi|17029714	46731	Pocillopora damicornis	84.15	164	23	3	491	652	280	442	2.00E-37	156
bu_91849.1_lrc26991	gi|17029716	46731	Pocillopora damicornis	84.15	164	23	3	491	652	280	442	2.00E-37	156
bu_91849.1_lrc26991	gi|17029716	46732	Pocillopora meandrina	84.15	164	23	3	491	652	280	442	2.00E-37	156
bu_91849.1_lrc26991	gi|17029717	46732	Pocillopora meandrina	84.15	164	23	3	491	652	280	442	2.00E-37	156
bu_91849.1_lrc26991	gi|17029717	512010	Pocillopora ligulata	84.15	164	23	3	491	652	280	442	2.00E-37	156
bu_91849.1_lrc26991	gi|17029718	512010	Pocillopora ligulata	84.15	164	23	3	491	652	280	442	2.00E-37	156
bu_91849.1_lrc26991	gi|17029718	512010	Pocillopora ligulata	84.15	164	23	3	491	652	280	442	2.00E-37	156
bu_91849.1_lrc26991	gi|17029718	512010	Pocillopora ligulata	84.15	164	23	3	491	652	280	442	2.00E-37	156
bu_91849.1_lrc26991	gi|17029718	512010	Pocillopora ligulata	84.15	164	23	3	491	652	280	442	2.00E-37	156
bu_91849.1_lrc26991	gi|17029718	512010	Pocillopora ligulata	84.15	164	23	3	491	652	280	442	2.00E-37	156
bu_91849.1_lrc26991	gi|17029718	367768	Pocillopora eydouxi	84.15	164	23	3	491	652	266	428	2.00E-37	156
bu_91849.1_lrc26991	gi|17029717	512010	Pocillopora ligulata	83.54	164	24	3	491	652	280	442	8.00E-36	150
bu_91849.1_lrc26991	gi|17029718	512010	Pocillopora ligulata	83.54	164	24	3	491	652	280	442	8.00E-36	150
bu_91849.1_lrc26991	gi|17029716	46732	Pocillopora meandrina	83.03	165	23	4	491	652	281	443	4.00E-34	145
bu_91849.1_lrc26991	gi|17029716	46732	Pocillopora meandrina	83.03	165	23	4	491	652	281	443	4.00E-34	145
bu_91849.1_lrc26991	gi|17029717	46732	Pocillopora meandrina	83.03	165	23	4	491	652	281	443	4.00E-34	145
bu_91849.1_lrc26991	gi|17029717	46732	Pocillopora meandrina	83.03	165	23	4	491	652	281	443	4.00E-34	145
bu_91849.1_lrc26991	gi|17029718	367768	Pocillopora eydouxi	83.03	165	23	4	491	652	281	443	4.00E-34	145
bu_91849.1_lrc26991	gi|17029718	367768	Pocillopora eydouxi	83.03	165	23	4	491	652	281	443	4.00E-34	145
bu_91849.1_lrc26991	gi|17029718	367768	Pocillopora eydouxi	82.93	164	24	4	491	652	262	423	4.00E-34	145
bu_91849.1_lrc26991	gi|17029719	367768	Pocillopora eydouxi	83.03	165	23	4	491	652	281	443	4.00E-34	145
bu_91849.1_lrc26991	gi|17029719	367768	Pocillopora eydouxi	82.93	164	24	4	491	652	262	423	4.00E-34	145
bu_91849.1_lrc26991	gi|17029719	512011	Pocillopora molokensis	82.93	164	24	4	491	652	262	423	4.00E-34	145
bu_91849.1_lrc26991	gi|17029719	512011	Pocillopora molokensis	82.93	164	24	4	491	652	262	423	4.00E-34	145
bu_91849.1_lrc26991	gi|17029719	512011	Pocillopora molokensis	82.93	164	24	4	491	652	262	423	4.00E-34	145
bu_91849.1_lrc26991	gi|17029720	512011	Pocillopora molokensis	82.93	164	24	4	491	652	262	423	4.00E-34	145
bu_91849.1_lrc26991	gi|17029712	46731	Pocillopora damicornis	82.32	164	25	4	491	652	262	423	2.00E-32	139
bu_91849.1_lrc26991	gi|17029713	46731	Pocillopora damicornis	82.32	164	25	4	491	652	262	423	2.00E-32	139
bu_91849.1_lrc26991	gi|17029713	46731	Pocillopora damicornis	82.32	164	25	4	491	652	280	441	2.00E-32	139
bu_91849.1_lrc26991	gi|17029713	46731	Pocillopora damicornis	82.32	164	25	4	491	652	262	423	2.00E-32	139
bu_91849.1_lrc26991	gi|17029713	46731	Pocillopora damicornis	82.32	164	25	4	491	652	280	441	2.00E-32	139

bu_91849.1_lrc26991	gi|17029713	46731	Pocillopora damicornis	82.32	164	25	4	491	652	262	423	2.00E-32	139
bu_91849.1_lrc26991	gi|17029714	46731	Pocillopora damicornis	82.32	164	25	4	491	652	262	423	2.00E-32	139
bu_91849.1_lrc26991	gi|17029714	46731	Pocillopora damicornis	82.32	164	25	4	491	652	262	423	2.00E-32	139
bu_91849.1_lrc26991	gi|17029714	46731	Pocillopora damicornis	82.32	164	25	4	491	652	262	423	2.00E-32	139
bu_91849.1_lrc26991	gi|17029714	46731	Pocillopora damicornis	82.32	164	25	4	491	652	262	423	2.00E-32	139
bu_91849.1_lrc26991	gi|17029715	46731	Pocillopora damicornis	82.32	164	25	4	491	652	262	423	2.00E-32	139
bu_91849.1_lrc26991	gi|17029715	46731	Pocillopora damicornis	82.32	164	25	4	491	652	262	423	2.00E-32	139
bu_91849.1_lrc26991	gi|17029715	46731	Pocillopora damicornis	82.32	164	25	4	491	652	262	423	2.00E-32	139
bu_91849.1_lrc26991	gi|17029715	46731	Pocillopora damicornis	82.32	164	25	4	491	652	262	423	2.00E-32	139
bu_91849.1_lrc26991	gi|17029715	46731	Pocillopora damicornis	82.32	164	25	4	491	652	262	423	2.00E-32	139
bu_91849.1_lrc26991	gi|17029716	46731	Pocillopora damicornis	82.32	164	25	4	491	652	262	423	2.00E-32	139
bu_91849.1_lrc26991	gi|17029716	46731	Pocillopora damicornis	82.32	164	25	4	491	652	262	423	2.00E-32	139
bu_91849.1_lrc26991	gi|17029719	512011	Pocillopora molokensis	83.89	149	21	3	491	638	262	408	2.00E-32	139
bu_91849.1_lrc26991	gi|17029719	512011	Pocillopora molokensis	83.89	149	21	3	491	638	262	408	2.00E-32	139
bu_91849.1_lrc26991	gi|17029719	512011	Pocillopora molokensis	83.89	149	21	3	491	638	262	408	2.00E-32	139
bu_91849.1_lrc26991	gi|17029719	512011	Pocillopora molokensis	83.89	149	21	3	491	638	262	408	2.00E-32	139
bu_91849.1_lrc26991	gi|17029719	512011	Pocillopora molokensis	83.89	149	21	3	491	638	262	408	2.00E-32	139
bu_91849.1_lrc26991	gi|17029720	512011	Pocillopora molokensis	83.89	149	21	3	491	638	262	408	2.00E-32	139
bu_91849.1_c27484	gi|15208488	48499	Montastraea franksi	94.25	1409	49	30	18	1417	1176	2561	0	2126
bu_91849.1_c27484	gi|16750770	46732	Pocillopora meandrina	98.76	161	1	1	18	178	731	890	4.00E-76	285
bu_91849.1_c27484	gi|16750776	46731	Pocillopora damicornis	98.76	161	1	1	18	178	731	890	4.00E-76	285
bu_91849.1_c27484	gi|16750770	123771	Madracis mirabilis	89.44	161	9	5	18	178	728	880	2.00E-49	196
bu_91849.1_c27484	gi|16750773	123773	Madracis senaria	89.44	161	9	5	18	178	728	880	2.00E-49	196
bu_91849.1_c27484	gi|16750774	123771	Madracis mirabilis	89.44	161	9	5	18	178	728	880	2.00E-49	196
bu_91849.1_c27484	gi|16750777	123769	Madracis decactis	89.44	161	9	5	18	178	728	880	2.00E-49	196
bu_91849.1_c27484	gi|16750768	123770	Madracis formosa	88.82	161	10	4	18	178	728	880	1.00E-47	191
bu_91849.1_c27484	gi|16750769	498791	Montastraea sp. MSB-2008	88.89	162	9	8	18	178	734	887	1.00E-47	191
bu_91849.1_c27484	gi|16750771	123769	Madracis decactis	88.82	161	10	4	18	178	728	880	1.00E-47	191
bu_91849.1_c27484	gi|16750773	123772	Madracis pharensis	88.82	161	10	5	18	178	728	880	1.00E-47	191
bu_91849.1_c27484	gi|16750774	465042	Madracis carmabi	88.82	161	10	5	18	178	728	880	1.00E-47	191
bu_91849.1_c27484	gi|16750776	214982	Mussa angulosa	88.89	162	9	8	18	178	734	887	1.00E-47	191
bu_91849.1_c27484	gi|16750773	46758	Lobophyllia hemprichii	88.82	161	9	7	18	178	728	879	3.00E-47	189
bu_91849.1_c27484	gi|16750767	214984	Mycetophyllia lamarckiana	88.27	162	11	7	18	178	734	888	1.00E-46	187
bu_91849.1_c27484	gi|16750770	63558	Montastraea cavernosa	88.27	162	11	8	18	178	728	882	1.00E-46	187
bu_91849.1_c27484	gi|16750775	130057	Crispatotrochus rugosus	88.27	162	12	6	18	178	727	882	1.00E-46	187
bu_91849.1_c27484	gi|22255061	45264	Acropora millepora	81.35	252	24	16	621	857	1	244	2.00E-45	183
bu_91849.1_c27484	gi|16750767	130053	Caryophyllia inornata	87.65	162	13	6	18	178	727	882	6.00E-45	182
bu_91849.1_c27484	gi|16750773	465062	Goniastrea retiformis	87.12	163	16	5	18	178	730	889	2.00E-44	180
bu_91849.1_c27484	gi|16750770	130191	Stephanocoenia michelinii	87.04	162	12	9	18	178	737	890	1.00E-42	174
bu_91849.1_c27484	gi|16750770	242734	Mycetophyllia aliciae	87.04	162	12	8	18	178	734	887	1.00E-42	174
bu_91849.1_c27484	gi|16750776	130191	Stephanocoenia michelinii	87.04	162	12	9	18	178	737	890	1.00E-42	174
bu_91849.1_c27484	gi|16750773	498799	Plesiastrea sp. MSB-2008	87.04	162	10	6	18	178	728	879	3.00E-42	172
bu_91849.1_c27484	gi|16750775	242718	Colpophyllia natans	86.5	163	14	7	18	178	745	901	3.00E-42	172
bu_91849.1_c27484	gi|16750767	75303	Heliofungia actiniformis	86.34	161	13	4	18	178	728	879	2.00E-40	167
bu_91849.1_c27484	gi|16750770	498788	Herpolitha sp. MSB-2008	86.34	161	13	4	18	178	728	879	2.00E-40	167
bu_91849.1_c27484	gi|16750772	498785	Coscinaraea sp. MSB-2008	86.34	161	13	4	18	178	728	879	2.00E-40	167
bu_91849.1_c27484	gi|16750775	498801	Sandalolitha sp. MSB-2008	86.34	161	13	4	18	178	728	879	2.00E-40	167
bu_91849.1_c27484	gi|16750769	498787	Halomitra sp. MSB-2008	85.71	161	14	5	18	178	728	879	8.00E-39	161
bu_91849.1_c27484	gi|16750774	46714	Lobactis scutaria	85.71	161	14	5	18	178	728	879	8.00E-39	161
bu_91849.1_c27484	gi|16750776	46717	Zoopilus echinatus	85.8	162	12	6	18	178	728	879	8.00E-39	161
bu_91849.1_c27484	gi|16750777	86586	Ceratotrochus magnaghii	85.19	162	18	6	18	178	749	905	8.00E-39	161

bu_91849.1_c27484	gi|16750777	498800	Polyphyllia sp. MSB-2008	85.8	162	12	6	18	178	728	879	8.00E-39	161
bu_91849.1_c27484	gi|16750777	46714	Lobactis scutaria	85.71	161	14	5	18	178	728	879	8.00E-39	161
bu_91849.1_c27484	gi|16750767	48498	Montastraea faveolata	85.71	161	11	7	18	178	728	876	3.00E-38	159
bu_91849.1_c27484	gi|16750771	63558	Montastraea cavernosa	88.41	138	9	7	18	154	728	859	3.00E-38	159
bu_91849.1_c27484	gi|16750772	465063	Cynarina lacrimalis	85.71	161	11	9	18	178	728	876	3.00E-38	159
bu_91849.1_c27484	gi|16750774	48499	Montastraea franksi	85.71	161	11	7	18	178	728	876	3.00E-38	159
bu_91849.1_c27484	gi|16750777	498796	Oxypora sp. MSB-2008	85.37	164	11	10	18	178	728	881	1.00E-37	158
bu_91849.1_c27484	gi|16750773	498797	Pachyseris sp. MSB-2008	84.76	164	15	8	18	178	738	894	4.00E-37	156
bu_91849.1_c27484	gi|16750770	498795	Oulophyllia sp. MSB-2008	84.66	163	15	8	18	178	730	884	1.00E-36	154
bu_91849.1_c27484	gi|16750775	46740	Hydnophora rigida	84.76	164	11	10	18	178	727	879	5.00E-36	152
bu_91849.1_c27484	gi|16750774	130199	Anthemiphyllia spinifera	87.05	139	9	7	18	154	729	860	6.00E-35	148
bu_91849.1_c27484	gi|16750769	498813	Stephanocyathus weberianus	83.54	164	15	11	18	178	735	889	3.00E-33	143
bu_91849.1_c27484	gi|16750772	465075	Agaricia grahamae	82.74	168	15	11	18	178	752	912	1.00E-31	137
bu_91849.1_c27484	gi|16750767	465077	Gardineroseris planulata	82.82	163	14	11	18	178	742	892	2.00E-30	134
bu_91849.1_c27484	gi|22278592	45264	Acropora millepora	96.25	80	2	1	1284	1363	682	604	2.00E-29	130
bu_91849.1_c27484	gi|16750767	262284	Agaricia lamarcki	82.25	169	13	15	18	178	752	911	2.00E-29	130
bu_91849.1_c27484	gi|16750774	46726	Pavona varians	82.21	163	15	11	18	178	742	892	8.00E-29	128
bu_91849.1_c27484	gi|16750768	214956	Agaricia undata	81.66	169	14	14	18	178	753	912	1.00E-27	124
bu_91849.1_c27484	gi|16750768	367765	Agaricia humilis	80.24	167	19	12	18	178	745	903	2.00E-24	113
bu_91849.1_c27484	gi|16750768	89882	Agaricia agaricites	79.88	169	19	13	18	178	745	906	3.00E-23	110
bu_91849.1_c27484	gi|16750775	89882	Agaricia agaricites	79.88	169	19	13	18	178	745	906	3.00E-23	110
bu_91849.1_c27484	gi|16750772	262285	Agaricia tenuifolia	78.7	169	21	13	18	178	745	906	6.00E-20	99
bu_91849.1_c27484	gi|16750776	498790	Leptoseris sp. MSB-2008	78.31	166	19	15	18	178	743	896	1.00E-17	91.6
bu_91849.1_lrc31664	gi|17029716	46732	Pocillopora meandrina	85.07	67	6	4	131	195	420	356	2.00E-10	65.8
bu_91849.1_lrc31664	gi|17029716	46732	Pocillopora meandrina	85.07	67	6	4	131	195	420	356	2.00E-10	65.8
bu_91849.1_lrc31664	gi|17029717	46732	Pocillopora meandrina	85.07	67	6	4	131	195	420	356	2.00E-10	65.8
bu_91849.1_lrc31664	gi|17029717	46732	Pocillopora meandrina	85.07	67	6	4	131	195	420	356	2.00E-10	65.8
bu_91849.1_lrc31664	gi|17029717	46732	Pocillopora meandrina	85.07	67	6	4	131	195	420	356	2.00E-10	65.8
bu_91849.1_lrc31664	gi|17029712	46731	Pocillopora damicornis	83.82	68	5	5	131	195	420	356	1.00E-08	60.2
bu_91849.1_lrc31664	gi|17029712	46731	Pocillopora damicornis	83.82	68	5	5	131	195	420	356	1.00E-08	60.2
bu_91849.1_lrc31664	gi|17029713	46731	Pocillopora damicornis	83.82	68	5	5	131	195	420	356	1.00E-08	60.2
bu_91849.1_lrc31664	gi|17029713	46731	Pocillopora damicornis	83.82	68	5	5	131	195	420	356	1.00E-08	60.2
bu_91849.1_lrc31664	gi|17029713	46731	Pocillopora damicornis	83.82	68	5	5	131	195	420	356	1.00E-08	60.2
bu_91849.1_lrc31664	gi|17029713	46731	Pocillopora damicornis	83.82	68	5	5	131	195	420	356	1.00E-08	60.2
bu_91849.1_lrc31664	gi|17029713	46731	Pocillopora damicornis	83.82	68	5	5	131	195	420	356	1.00E-08	60.2
bu_91849.1_lrc31664	gi|17029714	46731	Pocillopora damicornis	83.82	68	5	5	131	195	420	356	1.00E-08	60.2
bu_91849.1_lrc31664	gi|17029714	46731	Pocillopora damicornis	83.82	68	5	5	131	195	466	402	1.00E-08	60.2
bu_91849.1_lrc31664	gi|17029714	46731	Pocillopora damicornis	83.82	68	5	5	131	195	420	356	1.00E-08	60.2
bu_91849.1_lrc31664	gi|17029714	46731	Pocillopora damicornis	83.82	68	5	5	131	195	420	356	1.00E-08	60.2
bu_91849.1_lrc31664	gi|17029714	46731	Pocillopora damicornis	83.82	68	5	5	131	195	466	402	1.00E-08	60.2
bu_91849.1_lrc31664	gi|17029714	46731	Pocillopora damicornis	83.82	68	5	5	131	195	420	356	1.00E-08	60.2
bu_91849.1_lrc31664	gi|17029715	46731	Pocillopora damicornis	83.82	68	5	5	131	195	466	402	1.00E-08	60.2
bu_91849.1_lrc31664	gi|17029715	46731	Pocillopora damicornis	83.82	68	5	5	131	195	466	402	1.00E-08	60.2
bu_91849.1_lrc31664	gi|17029716	46731	Pocillopora damicornis	83.82	68	5	5	131	195	466	402	1.00E-08	60.2
bu_91849.1_lrc31664	gi|17029716	46731	Pocillopora damicornis	83.82	68	5	5	131	195	420	356	1.00E-08	60.2
bu_91849.1_lrc31664	gi|17029716	46731	Pocillopora damicornis	83.82	68	5	5	131	195	466	402	1.00E-08	60.2
bu_91849.1_lrc31664	gi|17029717	512010	Pocillopora ligulata	83.82	68	5	5	131	195	420	356	1.00E-08	60.2
bu_91849.1_lrc31664	gi|17029718	512010	Pocillopora ligulata	83.82	68	5	5	131	195	420	356	1.00E-08	60.2
bu_91849.1_lrc31664	gi|17029718	512010	Pocillopora ligulata	83.82	68	5	5	131	195	420	356	1.00E-08	60.2
bu_91849.1_lrc31664	gi|17029718	512010	Pocillopora ligulata	83.82	68	5	5	131	195	420	356	1.00E-08	60.2
bu_91849.1_lrc31664	gi|17029718	512010	Pocillopora ligulata	83.82	68	5	5	131	195	420	356	1.00E-08	60.2

bu_91849.1_lrc31664	gi|17029718	512010	Pocillopora ligulata	83.82	68	5	5	131	195	420	356	1.00E-08	60.2
bu_91849.1_lrc31664	gi|17029718	367768	Pocillopora eydouxi	83.82	68	5	5	131	195	406	342	1.00E-08	60.2
bu_91849.1_lrc31664	gi|17029712	46731	Pocillopora damicornis	78.64	103	9	10	131	228	401	307	1.00E-07	56.5
bu_91849.1_lrc31664	gi|17029713	46731	Pocillopora damicornis	78.64	103	9	10	131	228	401	307	1.00E-07	56.5
bu_91849.1_lrc31664	gi|17029713	46731	Pocillopora damicornis	78.64	103	9	10	131	228	419	325	1.00E-07	56.5
bu_91849.1_lrc31664	gi|17029713	46731	Pocillopora damicornis	78.64	103	9	10	131	228	401	307	1.00E-07	56.5
bu_91849.1_lrc31664	gi|17029713	46731	Pocillopora damicornis	78.64	103	9	10	131	228	419	325	1.00E-07	56.5
bu_91849.1_lrc31664	gi|17029713	46731	Pocillopora damicornis	78.64	103	9	10	131	228	401	307	1.00E-07	56.5
bu_91849.1_lrc31664	gi|17029714	46731	Pocillopora damicornis	78.64	103	9	10	131	228	401	307	1.00E-07	56.5
bu_91849.1_lrc31664	gi|17029714	46731	Pocillopora damicornis	78.64	103	9	10	131	228	401	307	1.00E-07	56.5
bu_91849.1_lrc31664	gi|17029714	46731	Pocillopora damicornis	78.64	103	9	10	131	228	401	307	1.00E-07	56.5
bu_91849.1_lrc31664	gi|17029714	46731	Pocillopora damicornis	78.64	103	9	10	131	228	401	307	1.00E-07	56.5
bu_91849.1_lrc31664	gi|17029715	46731	Pocillopora damicornis	78.64	103	9	10	131	228	401	307	1.00E-07	56.5
bu_91849.1_lrc31664	gi|17029715	46731	Pocillopora damicornis	78.64	103	9	10	131	228	401	307	1.00E-07	56.5
bu_91849.1_lrc31664	gi|17029715	46731	Pocillopora damicornis	78.64	103	9	10	131	228	401	307	1.00E-07	56.5
bu_91849.1_lrc31664	gi|17029715	46731	Pocillopora damicornis	78.64	103	9	10	131	228	401	307	1.00E-07	56.5
bu_91849.1_lrc31664	gi|17029715	46731	Pocillopora damicornis	78.64	103	9	10	131	228	401	307	1.00E-07	56.5
bu_91849.1_lrc31664	gi|17029716	46731	Pocillopora damicornis	78.64	103	9	10	131	228	401	307	1.00E-07	56.5
bu_91849.1_lrc31664	gi|17029716	46731	Pocillopora damicornis	78.64	103	9	10	131	228	401	307	1.00E-07	56.5
bu_91849.1_lrc31664	gi|17029718	367768	Pocillopora eydouxi	78.64	103	9	10	131	228	401	307	1.00E-07	56.5
bu_91849.1_lrc31664	gi|17029719	367768	Pocillopora eydouxi	78.64	103	9	10	131	228	401	307	1.00E-07	56.5
bu_91849.1_lrc31664	gi|17029719	512011	Pocillopora molokensis	78.64	103	9	10	131	228	401	307	1.00E-07	56.5
bu_91849.1_lrc31664	gi|17029719	512011	Pocillopora molokensis	78.64	103	9	10	131	228	401	307	1.00E-07	56.5
bu_91849.1_lrc31664	gi|17029719	512011	Pocillopora molokensis	78.64	103	9	10	131	228	401	307	1.00E-07	56.5
bu_91849.1_lrc31664	gi|17029719	512011	Pocillopora molokensis	78.64	103	9	10	131	228	401	307	1.00E-07	56.5
bu_91849.1_lrc31664	gi|17029719	512011	Pocillopora molokensis	78.64	103	9	10	131	228	401	307	1.00E-07	56.5
bu_91849.1_lrc31664	gi|17029719	512011	Pocillopora molokensis	78.64	103	9	10	131	228	401	307	1.00E-07	56.5
bu_91849.1_lrc31664	gi|17029719	512011	Pocillopora molokensis	78.64	103	9	10	131	228	401	307	1.00E-07	56.5
bu_91849.1_lrc31664	gi|17029719	512011	Pocillopora molokensis	78.64	103	9	10	131	228	401	307	1.00E-07	56.5
bu_91849.1_lrc31664	gi|17029720	512011	Pocillopora molokensis	78.64	103	9	10	131	228	401	307	1.00E-07	56.5
bu_91849.1_lrc31664	gi|17029720	512011	Pocillopora molokensis	78.64	103	9	10	131	228	401	307	1.00E-07	56.5
bu_91849.1_lrc31664	gi|17029712	46731	Pocillopora damicornis	96.88	32	1	0	131	162	420	389	5.00E-07	54.7
bu_91849.1_c36270	gi|11935226	46731	Pocillopora damicornis	97.22	36	1	0	187	222	299	334	7.00E-10	63.9
bu_91849.1_c36858	gi|75238520	46731	Pocillopora damicornis	95.31	832	30	9	16	841	18	846	0	1336
bu_91849.1_c36858	gi|19958145	45264	Acropora millepora	78.38	703	96	47	87	752	1	684	1.00E-113	409
bu_91849.1_c36858	gi|49257064	258444	Acropora sp. #30	77.55	646	96	38	140	752	58	687	3.00E-95	348
bu_91849.1_c36858	gi|25905351	6127	Acropora	77.55	646	96	38	140	752	58	687	3.00E-95	348
bu_91849.1_c36858	gi|25905351	6127	Acropora	77.55	646	96	38	140	752	58	687	3.00E-95	348
bu_91849.1_c36858	gi|29503331	46712	Fungia	77.55	646	96	38	140	752	58	687	3.00E-95	348
bu_91849.1_c36858	gi|33261315	46712	Fungia	77.55	646	96	38	140	752	58	687	3.00E-95	348
bu_91849.1_c36858	gi|22278513	45264	Acropora millepora	84.95	279	34	7	243	517	43	317	4.00E-74	278
bu_91849.1_lrc48708	gi|30330618	51070	Seriatopora hystrix	92.16	1339	79	22	27	1354	1380	57	0	1869
bu_91849.1_lrc48708	gi|38176181	50429	Stylophora pistillata	85.55	1142	151	14	222	1356	1189	55	0	1182
bu_91849.1_lrc48708	gi|26522943	211865	Favites chinensis	89.64	927	92	4	276	1200	925	1	0	1177
bu_91849.1_lrc48708	gi|26522783	46745	Galaxea fascicularis	83.3	1144	167	23	222	1353	1217	86	0	1033
bu_91849.1_lrc48708	gi|86562561	75303	Heliofungia actiniformis	84.09	553	78	10	400	947	548	1	5.00E-155	547
bu_91849.1_lrc48708	gi|22280314	45264	Acropora millepora	87.77	466	52	5	221	683	466	3	2.00E-153	542
bu_91849.1_lrc48708	gi|25568962	46704	Montipora capitata	88.49	304	27	8	280	579	300	1	7.00E-99	361
bu_91849.1_lrc48708	gi|23881742	46731	Pocillopora damicornis	98.46	195	3	0	1098	1292	196	2	7.00E-94	344
bu_91849.1_lrc48708	gi|23881742	46720	Porites compressa	96.92	195	6	0	1098	1292	196	2	7.00E-89	327
bu_91849.1_lrc48708	gi|22278857	45264	Acropora millepora	93.2	206	14	0	1039	1244	206	1	1.00E-81	303

bu_91849.1_lrc53513	gi|17029720	46731	Pocillopora damicornis	99.7	331	0	1	13	342	162	492	2.00E-172	604
bu_91849.1_lrc53513	gi|17029720	46731	Pocillopora damicornis	99.7	331	0	1	13	342	162	492	2.00E-172	604
bu_91849.1_lrc53513	gi|17029720	46731	Pocillopora damicornis	99.7	331	0	1	13	342	162	492	2.00E-172	604
bu_91849.1_lrc53513	gi|17029721	46731	Pocillopora damicornis	99.7	331	0	1	13	342	162	492	2.00E-172	604
bu_91849.1_lrc53513	gi|17029721	46731	Pocillopora damicornis	99.7	331	0	1	13	342	162	492	2.00E-172	604
bu_91849.1_lrc53513	gi|17029722	46731	Pocillopora damicornis	99.7	331	0	1	13	342	162	492	2.00E-172	604
bu_91849.1_lrc53513	gi|17029722	46731	Pocillopora damicornis	99.7	331	0	1	13	342	162	492	2.00E-172	604
bu_91849.1_lrc53513	gi|17029722	46731	Pocillopora damicornis	99.7	331	0	1	13	342	162	492	2.00E-172	604
bu_91849.1_lrc53513	gi|17029723	46731	Pocillopora damicornis	99.7	331	0	1	13	342	162	492	2.00E-172	604
bu_91849.1_lrc53513	gi|17029720	46731	Pocillopora damicornis	99.4	331	1	1	13	342	162	492	8.00E-171	599
bu_91849.1_lrc53513	gi|17029720	46731	Pocillopora damicornis	98.79	331	3	1	13	342	162	492	2.00E-167	588
bu_91849.1_lrc53513	gi|17029721	46731	Pocillopora damicornis	98.79	331	3	1	13	342	163	493	2.00E-167	588
bu_91849.1_lrc53513	gi|17029722	46731	Pocillopora damicornis	98.79	331	3	1	13	342	162	492	2.00E-167	588
bu_91849.1_lrc53513	gi|17029722	46731	Pocillopora damicornis	98.79	331	3	1	13	342	162	492	2.00E-167	588
bu_91849.1_lrc53513	gi|17029722	46731	Pocillopora damicornis	98.79	331	3	1	13	342	162	492	2.00E-167	588
bu_91849.1_lrc53513	gi|17029722	46731	Pocillopora damicornis	98.79	331	3	1	13	342	162	492	2.00E-167	588
bu_91849.1_lrc53513	gi|17029723	46731	Pocillopora damicornis	98.79	331	3	1	13	342	162	492	2.00E-167	588
bu_91849.1_lrc53513	gi|17029723	46731	Pocillopora damicornis	98.79	331	3	1	13	342	162	492	2.00E-167	588
bu_91849.1_lrc53513	gi|17029720	46731	Pocillopora damicornis	98.49	331	4	1	13	342	162	492	8.00E-166	582
bu_91849.1_lrc53513	gi|17029721	46731	Pocillopora damicornis	98.49	331	4	1	13	342	162	492	8.00E-166	582
bu_91849.1_lrc53513	gi|17029721	46731	Pocillopora damicornis	98.49	331	4	1	13	342	162	492	8.00E-166	582
bu_91849.1_lrc53513	gi|17029721	46731	Pocillopora damicornis	98.49	331	4	1	13	342	162	492	8.00E-166	582
bu_91849.1_lrc53513	gi|17029722	46731	Pocillopora damicornis	98.49	331	4	1	13	342	162	492	8.00E-166	582
bu_91849.1_lrc53513	gi|17029722	46731	Pocillopora damicornis	98.49	331	4	1	13	342	162	492	8.00E-166	582
bu_91849.1_lrc53513	gi|17029723	46731	Pocillopora damicornis	98.49	331	4	1	13	342	162	492	8.00E-166	582
bu_91849.1_lrc53513	gi|17029726	512011	Pocillopora molokensis	98.49	331	4	1	13	342	162	492	8.00E-166	582
bu_91849.1_lrc53513	gi|17029727	512011	Pocillopora molokensis	98.49	331	4	1	13	342	162	492	8.00E-166	582
bu_91849.1_lrc53513	gi|17029727	512011	Pocillopora molokensis	98.49	331	4	1	13	342	162	492	8.00E-166	582
bu_91849.1_lrc53513	gi|17029723	46732	Pocillopora meandrina	98.19	331	5	1	13	342	162	492	4.00E-164	577
bu_91849.1_lrc53513	gi|17029724	46732	Pocillopora meandrina	98.19	331	5	1	13	342	162	492	4.00E-164	577
bu_91849.1_lrc53513	gi|17029725	46732	Pocillopora meandrina	98.19	331	5	1	13	342	162	492	4.00E-164	577
bu_91849.1_lrc53513	gi|17029723	46732	Pocillopora meandrina	97.89	332	5	2	13	342	161	492	5.00E-163	573
bu_91849.1_lrc53513	gi|17029724	46732	Pocillopora meandrina	97.89	332	5	2	13	342	161	492	5.00E-163	573
bu_91849.1_lrc53513	gi|17029724	46732	Pocillopora meandrina	97.89	332	5	2	13	342	161	492	5.00E-163	573
bu_91849.1_lrc53513	gi|17029720	46731	Pocillopora damicornis	97.89	331	6	1	13	342	162	492	2.00E-162	571
bu_91849.1_lrc53513	gi|17029723	46732	Pocillopora meandrina	97.89	331	6	1	13	342	162	492	2.00E-162	571
bu_91849.1_lrc53513	gi|17029724	46732	Pocillopora meandrina	97.89	331	6	1	13	342	162	492	2.00E-162	571
bu_91849.1_lrc53513	gi|17029726	367768	Pocillopora eydouxi	97.58	331	5	2	13	342	162	490	3.00E-160	564
bu_91849.1_lrc53513	gi|17029724	46732	Pocillopora meandrina	96.98	331	9	1	13	342	165	495	2.00E-157	555
bu_91849.1_lrc53513	gi|17029726	367768	Pocillopora eydouxi	96.98	331	9	1	13	342	166	496	2.00E-157	555
bu_91849.1_lrc53513	gi|17029721	46731	Pocillopora damicornis	96.68	331	10	1	13	342	162	492	8.00E-156	549
bu_91849.1_lrc53513	gi|17029724	46732	Pocillopora meandrina	92.22	334	22	3	13	342	81	414	7.00E-132	470
bu_91849.1_lrc53513	gi|17029724	46732	Pocillopora meandrina	92.22	334	22	3	13	342	81	414	7.00E-132	470
bu_91849.1_lrc53513	gi|17029724	46732	Pocillopora meandrina	92.22	334	22	3	13	342	81	414	7.00E-132	470
bu_91849.1_lrc53513	gi|17029726	512011	Pocillopora molokensis	91.94	335	21	5	13	342	162	495	3.00E-130	464
bu_91849.1_lrc53513	gi|17029727	512011	Pocillopora molokensis	91.94	335	21	5	13	342	162	495	3.00E-130	464
bu_91849.1_lrc53513	gi|17029727	512011	Pocillopora molokensis	91.94	335	21	5	13	342	162	495	3.00E-130	464
bu_91849.1_lrc53513	gi|17029723	46732	Pocillopora meandrina	95.9	244	6	4	103	342	119	362	1.00E-108	392
bu_91849.1_lrc53513	gi|17029723	46732	Pocillopora meandrina	95.9	244	6	4	103	342	119	362	1.00E-108	392
bu_91849.1_lrc53513	gi|17029724	46732	Pocillopora meandrina	95.9	244	6	4	103	342	119	362	1.00E-108	392
bu_91849.1_lrc53513	gi|17029724	46732	Pocillopora meandrina	95.9	244	6	4	103	342	119	362	1.00E-108	392

bu_91849.1_lrc53513	gi|89994687	46731	Pocillopora damicornis	99.07	214	2	0	358	571	404	617	2.00E-106	385
bu_91849.1_lrc53513	gi|17029726	367768	Pocillopora eydouxi	93.55	217	8	5	131	342	487	702	3.00E-86	318
bu_91849.1_lrc53513	gi|17029720	46731	Pocillopora damicornis	92.66	218	9	6	131	342	470	686	5.00E-83	307
bu_91849.1_lrc53513	gi|17029721	46731	Pocillopora damicornis	92.63	217	10	5	131	342	470	685	5.00E-83	307
bu_91849.1_lrc53513	gi|17029722	46731	Pocillopora damicornis	92.63	217	10	5	131	342	488	703	5.00E-83	307
bu_91849.1_lrc53513	gi|17029723	46731	Pocillopora damicornis	92.63	217	10	5	131	342	488	703	5.00E-83	307
bu_91849.1_lrc53513	gi|17029726	512011	Pocillopora molokensis	92.63	217	10	5	131	342	488	703	5.00E-83	307
bu_91849.1_lrc53513	gi|17029726	512011	Pocillopora molokensis	92.63	217	10	5	131	342	488	703	5.00E-83	307
bu_91849.1_lrc53513	gi|17029727	512011	Pocillopora molokensis	92.63	217	10	5	131	342	488	703	5.00E-83	307
bu_91849.1_lrc53513	gi|17029727	512011	Pocillopora molokensis	92.63	217	10	5	131	342	472	687	5.00E-83	307
bu_91849.1_lrc53513	gi|17029721	46731	Pocillopora damicornis	92.17	217	11	5	131	342	486	701	3.00E-81	302
bu_91849.1_lrc53513	gi|17029725	512010	Pocillopora ligulata	92.17	217	11	5	131	342	486	701	3.00E-81	302
bu_91849.1_lrc53513	gi|17029725	512010	Pocillopora ligulata	92.17	217	11	5	131	342	488	703	3.00E-81	302
bu_91849.1_lrc53513	gi|17029725	512010	Pocillopora ligulata	92.17	217	11	5	131	342	488	703	3.00E-81	302
bu_91849.1_lrc53513	gi|17029725	512010	Pocillopora ligulata	92.17	217	11	5	131	342	488	703	3.00E-81	302
bu_91849.1_lrc53513	gi|17029725	512010	Pocillopora ligulata	92.17	217	11	5	131	342	488	703	3.00E-81	302
bu_91849.1_lrc53513	gi|17029726	512010	Pocillopora ligulata	92.17	217	11	5	131	342	488	703	3.00E-81	302
bu_91849.1_lrc53513	gi|17029726	512010	Pocillopora ligulata	92.17	217	11	5	131	342	488	703	3.00E-81	302
bu_91849.1_lrc53513	gi|17029726	512010	Pocillopora ligulata	92.17	217	11	5	131	342	488	703	3.00E-81	302
bu_91849.1_lrc53513	gi|17029725	512010	Pocillopora ligulata	91.71	217	12	5	131	342	488	703	1.00E-79	296
bu_91849.1_lrc53513	gi|17029725	512010	Pocillopora ligulata	91.71	217	12	5	131	342	488	703	1.00E-79	296
bu_91849.1_lrc53513	gi|17029725	512010	Pocillopora ligulata	91.71	217	12	5	131	342	488	703	1.00E-79	296
bu_91849.1_lrc53513	gi|17029721	46731	Pocillopora damicornis	91.71	217	11	6	131	342	488	702	4.00E-79	294
bu_91849.1_lrc53513	gi|17029725	512010	Pocillopora ligulata	91.71	217	11	6	131	342	488	702	4.00E-79	294
