# Supplementary material for: Production of a reference transcriptome and transcriptomic database (PocilloporaBase) for the cauliflower coral, Pocillopora damicornis
Source: BMC Genomics. 2011 Nov 29;12:585. doi: 10.1186/1471-2164-12-585 (PMC3339375; doi:10.1186/1471-2164-12-585)
Supplement: Additional file 8 — A diagram depicting KEGG pathway elements and whether they were found or not found among the P. damicornis contigs. [file 1471-2164-12-585-S8.PPT]

## Slide 1
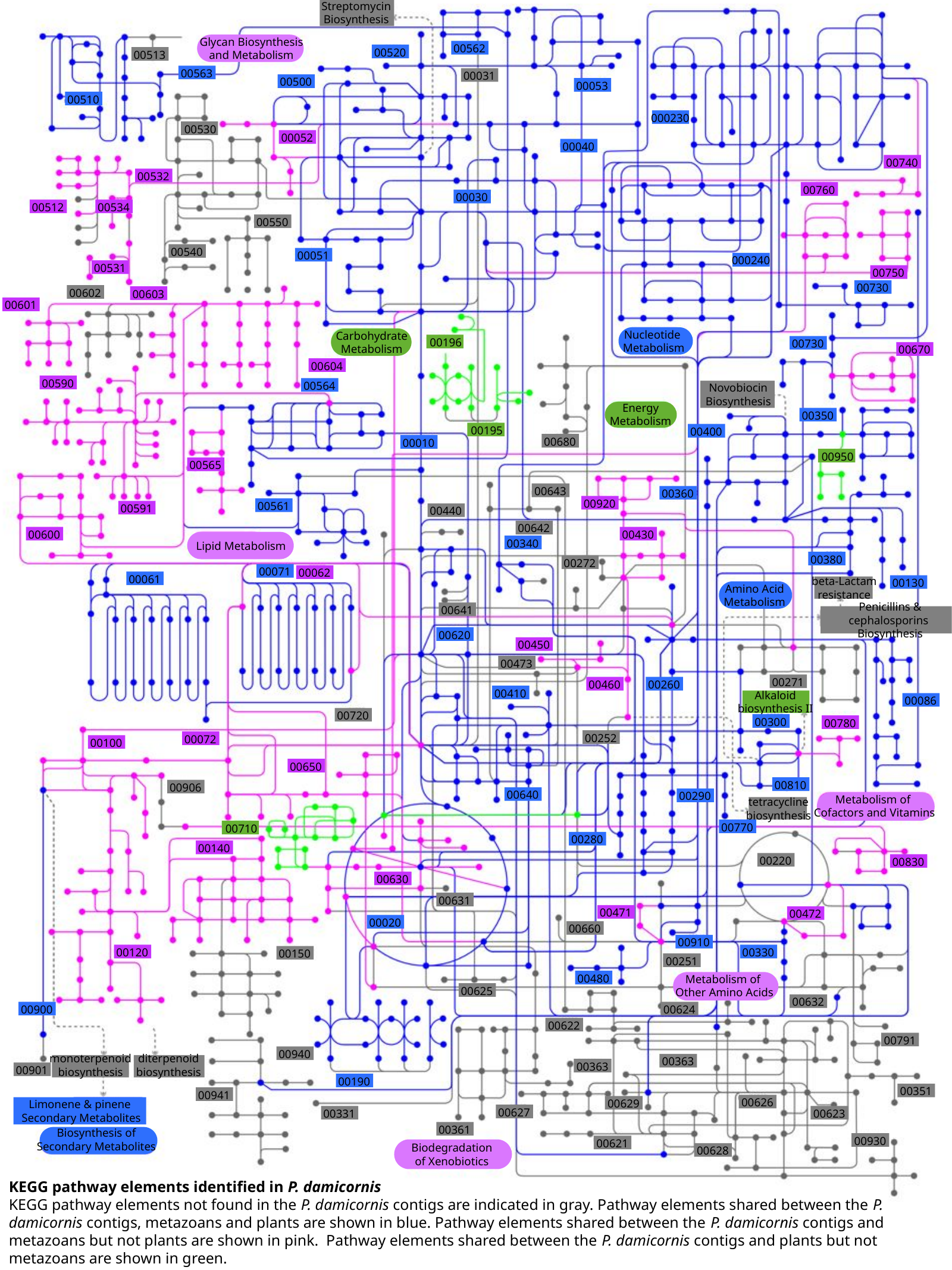

Streptomycin
Biosynthesis
Glycan Biosynthesis
and Metabolism
00562
00520
00513
00563
00031
00500
00053
00510
000230
00530
00052
00040
00740
00532
00760
00030
00512
00534
00550
00540
00051
000240
00531
00750
00730
00602
00603
00601
Nucleotide
Metabolism
Carbohydrate
Metabolism
00196
00730
00670
00604
00590
00564
Novobiocin
Biosynthesis
Energy
Metabolism
00350
00195
00400
00680
00010
00950
00565
00643
00360
00920
00561
00591
00440
00642
00600
00430
00340
Lipid Metabolism
00380
00272
00071
00062
beta-Lactam
resistance
00061
00130
Amino Acid
Metabolism
00641
Penicillins & cephalosporins
Biosynthesis
00620
00450
00473
00271
00460
00260
Alkaloid
biosynthesis II
00410
00086
00720
00300
00780
00252
00072
00100
00650
00810
00906
00640
00290
tetracycline
biosynthesis
Metabolism of
Cofactors and Vitamins
00770
00710
00280
00140
00220
00830
00630
00631
00471
00472
00020
00660
00910
00120
00330
00150
00251
00480
Metabolism of
Other Amino Acids
00625
00632
00624
00900
00622
00791
00940
monoterpenoid
biosynthesis
diterpenoid
biosynthesis
00363
00363
00901
00190
00351
00941
00626
00629
Limonene & pinene
Secondary Metabolites
00623
00627
00331
00361
Biosynthesis of
Secondary Metabolites
00930
00621
00628
Biodegradation
of Xenobiotics
Metabolism of
Cofactors and Vitamins
KEGG pathway elements identified in P. damicornis
KEGG pathway elements not found in the P. damicornis contigs are indicated in gray. Pathway elements shared between the P. damicornis contigs, metazoans and plants are shown in blue. Pathway elements shared between the P. damicornis contigs and metazoans but not plants are shown in pink. Pathway elements shared between the P. damicornis contigs and plants but not metazoans are shown in green.
