# Supplementary material for: Production of a reference transcriptome and transcriptomic database (PocilloporaBase) for the cauliflower coral, Pocillopora damicornis
Source: BMC Genomics. 2011 Nov 29;12:585. doi: 10.1186/1471-2164-12-585 (PMC3339375; doi:10.1186/1471-2164-12-585)
Supplement: Additional file 9 — A compressed folder containing an interactive (iPath) version of Additional File 7, which shows the metabolic pathways present in P. damicornis as inferred by BLASTx matches to human or plant genes with known metabolic functions. [file 1471-2164-12-585-S9.ZIP › rekeggfigure/iPath Interactive Pathways Explorer.html]

iPath: Interactive Pathways Explorer


iPath: Interactive Pathways Explorer

- HOME
- GLOBAL MAP
- ANALYSIS AND DATA MAPPING
- TAXONOMY RESTRICTED MAPS

## Global metabolic pathways map

Clicking on nodes or edges will display detailed information. Zoom in to increase the level of detail.

This map is displayed using Flash, and your browser does not have a proper plugin installed. You can download and install the plugin from Adobe Flash download page

Send comments to Ivica Letunic
